# SAMJ: fast image annotation on ImageJ/Fiji via segment anything model

**DOI:** 10.1038/s41467-026-71752-x

**Published:** 2026-06-18

**Authors:** Carlos García-López-de-Haro, Caterina Fuster-Barceló, Curtis T. Rueden, Jónathan Heras, Vladimír Ulman, Daniel Franco-Barranco, Adrián Inés, Kevin W. Eliceiri, Jean-Christophe Olivo-Marin, Jean-Yves Tinevez, Daniel Sage, Arrate Muñoz-Barrutia

**Affiliations:** 1 Neuroscience and Life Sciences Department, Universidad Carlos III de Madrid, Getafe, Spain; 2 Bioimage Analysis Unit, Institut Pasteur, Université Paris Cité, Paris, France; 3 Bioengineering Department, Universidad Carlos III de Madrid, Leganes, Spain; 4 BioVisionCenter, University of Zurich, Zurich, Switzerland; 5 Center for Quantitative Cell Imaging, University of Wisconsin, Madison, USA; 6 Department of Mathematics and Computer Science, University of La Rioja, Logroño, Spain; 7 Centre for Biomedical Image Analysis, Masaryk University, Brno, Czech Republic; 8 Central European Institute of Technology (CEITEC), Masaryk University, Brno, Czech Republic; 9 MRC Laboratory of Molecular Biology, Cambridge, UK; 10 Department of Physiology, Development and Neuroscience, University of Cambridge, Cambridge, UK; 11 Donostia International Physics Center (DIPC), San Sebastian, Spain; 12 CNRS UMR 3691, Institut Pasteur, Paris, France; 13 Faculty of Health Sciences, Universidad Carlos III de Madrid, Getafe, Spain; 14 Image Analysis Hub, Institut Pasteur, Université Paris Cité, Paris, France; 15 Biomedical Imaging Group and Center for Imaging, Ecole Polytechnique Fédérale de Lausanne (EPFL), Lausanne, Switzerland; 16 Bioengineering Division, Instituto de Investigación Sanitaria Gregorio Marañón, Madrid, Spain

**Keywords:** Image processing, Machine learning

## Abstract

Accurate image annotation is essential for training artificial intelligence (AI) systems in biomedical image analysis, enabling tasks such as cell detection, tissue quantification, and disease characterization. However, creating pixel-level annotations is a time-consuming and labor-intensive process that requires expert input, limiting the development and adoption of AI methods. Recent advances in foundation models, such as the Segment Anything Model (SAM), enable interactive object segmentation from simple user prompts, but their integration into widely used bioimage analysis platforms remains limited and often requires technical expertise. Here we show that SAMJ, a user-friendly plugin for ImageJ/Fiji, enables fast, interactive, and accurate image annotation on standard computers without requiring programming skills or specialized hardware. SAMJ integrates efficient SAM variants into a familiar graphical interface, allowing users to delineate objects in large scientific images in real time using simple clicks or bounding boxes. This approach significantly reduces annotation effort, accelerates dataset creation, and broadens access to advanced AI-assisted annotation tools for the biomedical research community.

## Abstract (web summary)

García-López-de-Haro and colleagues present SAMJ, a plugin that brings the Segment Anything AI to ImageJ/Fiji, enabling fast, click-based image annotation on standard computers, and accelerating creation of biomedical training datasets.

## Introduction

Access to high-quality annotated images is key to training and developing new methods for biomedical image analysis and object segmentation. However, creating large datasets of annotated images is a time-consuming, labor-intensive, and subjective process^1^. The demand becomes even more pronounced when training supervised Deep Learning (DL) models, typically requiring hundreds to thousands of annotated images^2^. Therefore, there is a critical need for efficient annotation tools capable of running on commonly available computers. Such tools must reduce IT-related barriers, ensuring broader accessibility for domain-specific experts such as biologists.

These solutions should also be compatible with widely used bioimage software platforms, such as ImageJ/Fiji^3^, and must be fast enough to accelerate the annotation of dozens of objects in large images, which are typical in biological research and essential for training machine learning models.

Recently, Meta Research released the Segment Anything Model (SAM)^4^, a foundational DL framework designed for one-shot and promptable image segmentation. SAM represents a significant breakthrough, capable of delineating complex objects and enabling semi-automated, interactive annotation across diverse image types. In its standard mode of operation, SAM requires user-defined prompts, such as points, sets of points, or bounding boxes, to efficiently isolate target objects.

SAM has been made available in commonly used bioimage software packages, such as QuPath^5^ and Napari^6^ (see section 16), providing interactive annotation, but has not yet been incorporated into the ImageJ ecosystem^7^. Other proprietary software, such as Matlab, ArcGIS, Kili Technology, or Unitlab Magic Touch have incorporated SAM as an add-on tool to enhance their segmentation capabilities. Similarly, Segment Anything Annotator, an open-source Python-based UI application, uses SAM for pixel-level annotation.

Although many annotation tools are both user-friendly and widely accessible, they often face challenges when dealing with uneven illumination, overlapping structures, or subtle intensity gradients. Several plugins have introduced more advanced methods: AnnotatorJ^8^, a popular Fiji plugin, supports semi-automatic cellular annotation by combining manual input with deep learning; Labkit^9^ leverages machine learning for pixel-wise classification; and Thomas et al.^10^ propose a streamlined approach to systematic manual annotation by assigning predefined categories to images or regions. However, none of these plugins yet benefit from the advantages that SAM provides.

In this manuscript, we present SAMJ, a plugin that brings the capabilities of SAM into the Fiji ecosystem, both as a standalone Fiji plugin and as an extension for Labkit^9^. We designed SAMJ for one-click installation. It features a no-code interface, and runs on standard computers without requiring specialized hardware. By lowering technical barriers, it broadens access to cutting-edge AI tools within the life sciences community. A key contribution of our work is the tailored adaptation of SAMJ to efficiently handle scientific images. By harnessing the powerful segmentation capabilities of SAM, this tool has the potential to significantly accelerate the annotation process, enabling users to instantly generate regions of interest around target objects with a single point click or bounding box.

## Results

### Design and architecture of the SAMJ plugin

It is important to note that SAMJ does not introduce a novel model for bioimage segmentation. Instead, it provides a software tool that facilitates the use of an existing foundational model (SAM), which we believe can be highly valuable for bioimage annotation, especially within Fiji where its environment, usability, and macro language make it an ideal setting for high-throughput annotation and automation.

SAMJ leverages the core architecture of SAM, which is built upon three key components: an image encoder, a prompt encoder, and a mask decoder. The image encoder is based on a Visual Transformer (ViT)^11^ that encodes the input image, offering high representational power at the expense of significant computational resources. The prompt encoder is another transformer to encode the prompt given by the user to point or hint to the location of the object of interest. Finally, the encoded image and prompt are used by the mask decoder, which combines elements of both a transformer and a Convolutional Neural Network, to generate the mask of interest.

### Accessibility and usability

The SAMJ plugin is installed as any standard Fiji extension, eliminating the technical IT barriers often associated with Python packages and making advanced image segmentation accessible to a wider audience. Unlike most Python-based libraries that require command-line interfaces for environment configuration and dependency management, SAMJ simplifies the process with a seamless, one-click installation, enabling effortless deployment.

In addition, SAMJ provides access to five SAM variants: SAM-2^12^ (Tiny, Small, and Large), EfficientSAM^13^, and EfficientViTSAM-L2^14^. All variants perform the same segmentation task and are trained on comparable generalist datasets, but they differ substantially in computational requirements and performance characteristics (see Supplementary Information section 1). This diversity enables users to select a model that fits both their hardware and application needs, ensuring that at least one option remains practical to run on any virtual-machine, workstation or mid-range laptop. For instance, users may prioritize speed and efficiency on less powerful machines or higher annotation accuracy on complex images.

### Integration with the Fiji ecosystem and Java–Python interoperability

Beyond its ease of use, SAMJ is designed for adaptability and seamless integration. Powered by SAM, it can enhance annotation workflows across a range of Java-based platforms, for example, in addition to Labkit, we have also integrated it into BigDataViewer^15^. Moreover, to support broader adoption, SAMJ provides a well-documented API, enabling developers to easily incorporate SAM functionality into their preferred Java software effortlessly. Furthermore, it includes a software-agnostic Java GUI, facilitating straightforward integration into any Java-based environment.

A key strength of SAMJ is its integration of Python methods into Java environments. This is achieved through Appose^16^ (see section 16), a Java package that allows Java and Python to run as separate processes yet communicate in real time. Leveraging Micromamba^17^, Appose also automates Python environment setup, totally eliminating manual configuration via the command line. As a result, SAMJ is remarkably user-friendly, granting effortless access to advanced methods.

### Interactive annotation workflow in Fiji

**Fig. 1:**
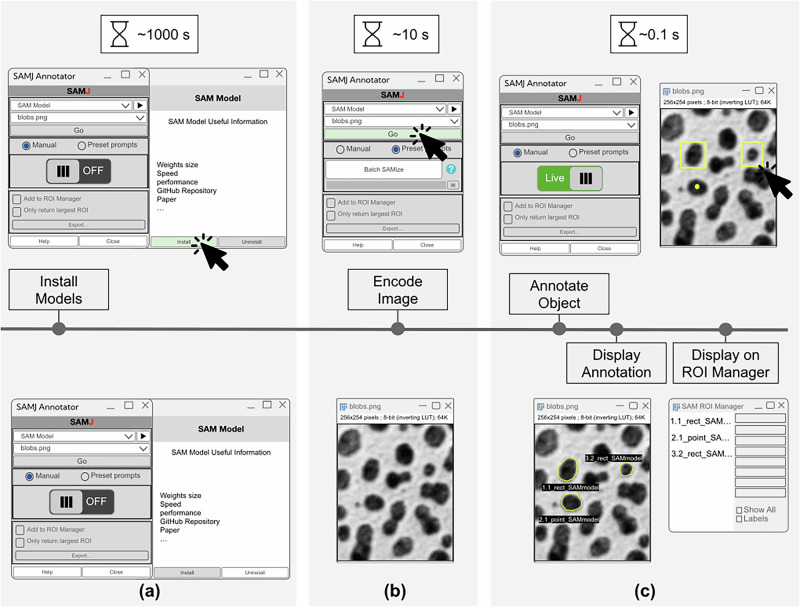
SAMJ User Interface and Workflow with estimated time. This figure illustrates the typical workflow of SAMJ for annotating objects in an image, including the time required for each step on standard workstations. **a** Model Installation: The most time-consuming step, where SAMJ installs the selected model and, if necessary, sets up the environment, taking approximately 1000 s. **b** Image Encoding: When the user clicks “Go” in the plugin, the image is processed to generate an embedding, which takes around 10 s. **c** Object Annotation: Since no re-embedding is required, this step is immediate, with each annotation generated in approximately 0.1 s per user click. The annotation process can be repeated multiple times as desired for different objects, offering rapid and interactive segmentation.

The annotation process in the SAMJ plugin mirrors SAM’s workflow (Fig. 1). Each image is encoded once by the image encoder, the most computationally intensive step. Afterwards, prompts are processed and masks generated almost instantly, allowing users to interactively produce multiple annotations with real-time responsiveness. SAMJ integrates seamlessly with Fiji’s existing tools, allowing users to create prompts such as points and rectangles directly through Fiji’s familiar toolbar, thereby minimizing the learning curve for new users.

The SAMJ plugin offers two annotation modes: Live and Batch mode (called BatchSAMize). In Live mode, prompts are drawn directly on the encoded image, and annotations are generated interactively, one at a time. In Batch mode, users can define multiple seed points to serve as prompts for segmenting multiple objects in a raw image. These seeds can be generated automatically using traditional Fiji commands (e.g., Find Maxima, Watershed) within an ImageJ macro, followed by a call to SAMJ’s Batch mode to segment the whole image. Additionally, users can enhance annotations by providing seeds from other segmentation methods as prompts, enabling iterative refinement. Prompts for BatchSAMize can be supplied either manually or automatically. Thanks to SAMJ’s integration with Fiji, users can combine bounding boxes or point prompts generated by deep learning models, macros, or files imported through the ROI Manager. Users can even use the macros to annotate batches of images with SAMJ, as illustrated in the Supplementary Materials. This flexibility enhances both the reproducibility and the automation of image annotation.

### Representative use cases

**Fig. 2:**
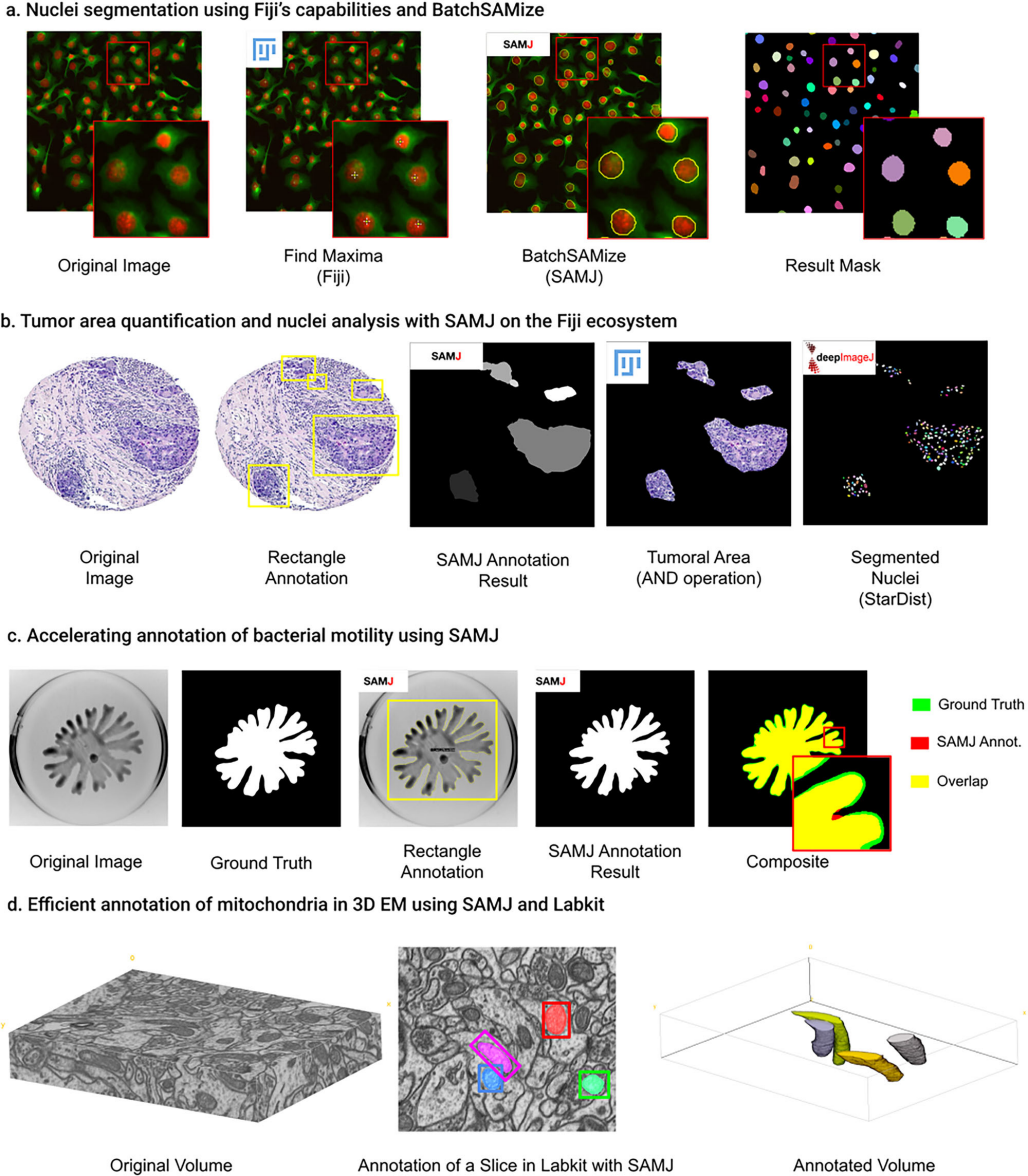
Use cases demonstrating the versatility of SAMJ for bioimage analysis. **a** Nuclei Segmentation using Fiji’s capabilities and BatchSAMize: An image from the CellPose dataset^21^ is used to demonstrate the segmentation of nuclei in the red channel. Pixel intensity maxima are identified in Fiji to generate single-point prompts for each nucleus. These prompts are processed in batch mode using SAMJ, resulting in semantic segmentation of individual nuclei. **b** Tumor Area Quantification and Nuclei Analysis with SAMJ and StarDist plugins for Fiji: Breast cancer TMA (Tissue Microarray) images, stained with H& E (Hematoxylin and Eosin) provided by the British Columbia Cancer Agency (BCCA)^28^, are used to quantify tumoral regions and their nuclei. Tumoral areas are annotated with SAMJ's rectangle prompt, generating masks that are combined with the original image in Fiji using an AND operation to obtain the intersection. StarDist is then applied through deepImageJ to segment individual nuclei in the tumoral areas. **c** Accelerating annotation of Bacterial Motility: SAMJ is applied to annotate motile bacteria using a single rectangle annotation. When compared to Ground Truth, SAMJ's annotations achieve comparable or superior precision, showcasing its efficiency in handling complex shapes and streamlining high-throughput workflows. Each use case highlights SAMJ's integration with Fiji, combining SAM's advanced annotation capabilities with Fiji’s extensive image processing tools. **d** Efficient annotation of 3D Electron Microscopy images of mitochondria^29^ using SAMJ and Labkit: SAMJ is integrated into Labkit to support 3D and multi-label annotation. Users can annotate structures on arbitrarily oriented and scaled slices, improving visibility and accuracy of objects with complex spatial orientation. The multi-class labeling capability of Labkit allows the annotation of several distinct structures within the same volume. This workflow reduces annotation effort and ensures spatial consistency across slices.

To illustrate the power of SAMJ and its seamless integration with the Fiji ecosystem, we present four representative use cases highlighting its capabilities (Fig. 2). These examples illustrate SAMJ’s ability to adapt to diverse image annotation tasks while taking advantage of Fiji’s powerful image processing tools. In all cases, the targets are compact and well-defined–conditions under which SAMJ performs particularly well. Elongated or branched structures may pose more of a challenge; However, users can refine prompts, adjust scale settings, or combine SAMJ with complementary methods. Experimentation is encouraged, as SAMJ often proves effective even under suboptimal imaging conditions (see section 16).

In the first use case, we illustrate nuclei segmentation in a fluorescence image using the BatchSAMize mode of SAMJ. Classical Fiji commands are used to detect nuclei, which serve as single-point prompts for batch processing in SAMJ. This workflow highlights the synergy between the preprocessing capabilities of Fiji and the segmentation power of SAMJ, streamlining large-scale annotation tasks.

The second use case intended for tumor area quantification focuses on breast cancer TMA images stained with H&E. The task involved quantifying tumoral areas and the number of nuclei within these regions. Using SAMJ’s rectangle annotation feature, tumoral regions were outlined, generating masks for these areas. Subsequently, individual nuclei were segmented with StarDist^18^ using the deepImageJ^7,19^ plugin. This workflow demonstrates SAMJ’s role as a flexible and efficient annotation tool that seamlessly integrates with Fiji, enabling efficient annotation and precise quantification for tumor analysis.

The third use case highlights SAMJ’s efficiency in annotating complex shapes^20^. Bacteria from motility studies were annotated using a single rectangle prompt per image, significantly reducing manual effort. When compared to manually generated ground truth, SAMJ’s annotations are highly consistent with the reference labels, as supported by the IoU results reported in Table 4. This use case demonstrates how SAMJ accelerates annotation tasks for intricate biological structures, making it a powerful tool for high-throughput studies.

The final use case demonstrates SAMJ’s capability for efficient annotation of mitochondria instances in 3D electron microscopy volumes through its integration with Labkit. Annotating objects in 3D is inherently challenging due to the difficulty of maintaining spatial coherence across slices and the substantial effort required for manual annotation. SAMJ streamlines this process, and its integration into Labkit–designed for interactive 3D visualization–enables users to annotate structures on arbitrarily oriented slices, ensuring optimal views of the object of interest. Moreover, Labkit support for multi-class labeling allows users to assign distinct labels to multiple structures within the same volume. This combination significantly reduces annotation effort while improving both accuracy and consistency, highlighting the power of integrating SAMJ with complementary Fiji tools for complex 3D bioimage annotation tasks. In this example, every slice of the volume was annotated with SAMJ, effectively following a pragmatic 2.5 D strategy. Thanks to SAMJ’s rapid interactive prompting, this approach still provided a substantial gain in speed compared to fully manual slice-by-slice annotation.

Together, these use cases highlight the versatility and strength of SAMJ integration within the Fiji ecosystem. By combining the advanced segmentation capabilities of SAM with the rich set of image processing tools of Fiji, SAMJ enables more efficient, precise, and scalable annotation workflows across a wide range of biological imaging tasks. This integration lowers technical barriers and supports diverse use cases, from 2D fluorescence images to complex 3D volumes. Consequently, it empowers the broader life science community–particularly biologists who primarily rely on GUI software platforms–with powerful deep-learning tools that would otherwise remain accessible only to a few, accelerating and improving their bioimage analysis.

### Annotating complex objects with multi-step prompting

The Segment Anything Model was originally trained with a one-prompt/one-object paradigm on a wide variety of objects, mostly from natural images. As a result, biological structures with intricate morphologies, such as neurons, are often difficult to annotate with a single prompt, regardless of prompt placement.

**Fig. 3:**
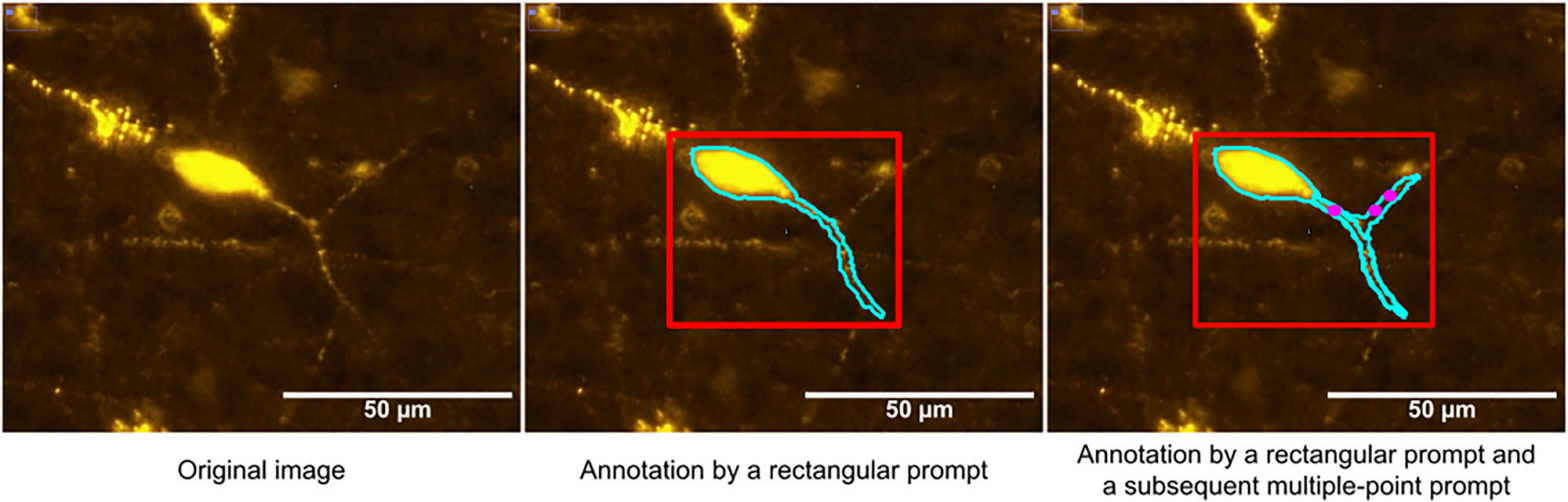
Multi-step annotation of a single object using different prompts. SAMJ's interactive workflow allows users to segment multiple parts of an object that the model recognizes and subsequently merge them. The figure shows a fluorescent neuron^30^ with two ramifications. Using a single box prompt (middle image), the model captures only one ramification. By annotating the second ramification with additional point prompts (purple dots on the right image), the user obtains two separate labels whose union yields the final mask of the complex neuron.

However, thanks to SAMJ interactivity, users can overcome this limitation by enabling a multi-step, compositional annotation workflow. While SAM may struggle to segment a branched structure globally, it remains highly effective at detecting local boundaries. SAMJ leverages this strength by allowing users to segment distinct sub-structures (e.g., soma, axons, and dendrites) individually. These partial masks can then be merged into a single, coherent object. This piecewise strategy yields significantly more accurate annotations for elongated or highly branched structures than single-prompt inference (see Fig. 3).

## Discussion

Accurate annotation is fundamental to deep learning-based image analysis, playing a critical role in model training, validation, and fine-tuning. To address the limitations posed by the high embedding latency of SAM, particularly in large microscopy images, we developed SAMJ, an intuitive and interactive annotation tool tailored for the life sciences. SAMJ integrates efficient SAM2 models optimized to run on mid-range computers and includes a Java-Python bridge to eliminate complex installation steps. As an ImageJ/Fiji plugin, it remains fully compatible with standard Fiji tools such as ROI selection, the ROI manager, and macros. Through this seamless integration, SAMJ enables efficient, high-quality annotations and supports the creation of curated datasets, thus contributing to the development of more accurate and robust models.

In conclusion, SAMJ provides a user-friendly and accessible way for biologists and bioimage analysts to adopt cutting-edge AI methods, facilitating faster and more precise image annotation and ultimately accelerating scientific discovery.

## Methods

### Ethics statement

This work describes a software tool and did not involve the recruitment of human participants, animal experimentation, or the collection of new identifiable personal data. All experiments were performed on publicly available datasets, used in accordance with their terms of use and licenses. Therefore, no additional institutional ethics committee/IRB approval was required for this study. This study complies with all relevant ethical regulations.

### Statistics and reproducibility

This study is primarily a software/methods report, and the main text is descriptive. Quantitative analyses were performed only in the comparative benchmarking experiments described in the Methods Section, in which SAMJ and two comparable tools were run on all images across the selected public datasets, and annotation performance (including IoU) were computed (Table 4). Sample sizes were determined by dataset availability and relevance. No statistical method was used to predetermine sample size. No data were excluded from the analyses. The experiments were not randomized. The Investigators were not blinded to allocation during experiments and outcome assessment. Reproducibility is supported by the use of publicly available datasets, reporting of software versions and benchmarking settings in the Methods Section, and release of the benchmarking code (https://github.com/segment-anything-models-java/benchmarking).

### Review of annotation tools for image segmentation

We provide a comprehensive overview of the existing annotation tools for bioimage analysis, summarizing key features, such as their release dates, supported annotation types, and integration with the Segment Anything Model (SAM) or any other AI assistant (Table 1). This table highlights the diversity of tools available across platforms like Fiji, QuPath, and Napari, as well as their varying levels of SAM integration. Notably, while tools such as QuPath and Napari have embraced SAM, Fiji-based plugins like AnnotatorJ lack direct SAM support. SAMJ addresses this gap by bringing SAM’s advanced annotation capabilities to the Fiji environment, combining flexibility with ease of use. This comparative analysis underscores SAMJ’s unique contribution to enabling SAM-powered annotation within the Fiji ecosystem.

**Table 1:** Summary of tools integrating annotation capabilities in bioimage analysis platforms

Tool/Plugin	Platform	Release	Last Update	AI assistance	Annotation	Programming language
MicroSAM	Napari	2023	03/2025	Yes (SAM fine-tune)	Polygons BBox Points	Python
QuPath Ext. SAM	QuPath	2023	09/2024	Yes (SAM2)	AutoMask BBox Poitns	Python
Napari Plugin of SAM	Napari	2023	04/2023	Yes (SAM1)	BBox Points	Python
Napari Plugin of SAM2	Napari	2024	09/2024	Yes (SAM2)	BBox Points	Python
AnnotatorJ	Fiji	2020	10/2020	Yes (CNN)	Instance Semantic BBox	Java
Labkit	Fiji	2022	10/2024	Yes (Random Forest SAM1,2)	Pixel Classification	Java
Qualitative Annotations	Fiji	2020	04/2021	Yes (CNN)	One or Multiple ROI	Python
SAMJ	Fiji	2025	04/2025	Yes (SAM1,2)	BBox Points	Java

The table highlights the main tools and plugins used for annotation in various bioimage analysis platforms, detailing their release (YYYY) and last update dates (MM/YYYY, Last accessed: 20 MAY 2025), annotation features, integration with the SAM, and the programming languages used for their development. SAM integration is specified with its supported annotation types, including bounding boxes (BBox), points, polygons, and more, showcasing the variety of annotation modes available.

### Review of SAM-based annotation tools for bioimaging

We have benchmarked the most widely used SAM-based bioimage annotation tools: SAMJ, Micro-SAM^6^, and the SAM extension for QuPath^5^. Each of these three tools serves a distinct niche, and their suitability depends on the user’s requirements. Tables 2, 3 summarize aspects related to ease of use (available models, CPU-only compatibility, optional GPU acceleration, model-loading time, prompt-response speed, and one-click installation). Table 4 reports the intersection-over-union (IoU) of annotations generated by each tool across multiple datasets.

**Table 2:** Comparison of features across SAM-based annotation tools

Software	One-Click	CPU	GPU	Models
	Installation	Support	Support	Available
SAMJ	YES	YES	NO	SAM2 Tiny
	YES	YES	NO	SAM2 Small
	YES	YES	NO	SAM2 Large
	YES	YES	NO	EfficientSAM
	YES	YES	NO	EfficientViTSAM-L2
Micro-SAM	NO	Slow	YES	SAM Base
	NO	Slow	YES	SAM Base Light
	NO	Slow	YES	SAM Base LM fine-tuned
	NO	Slow	YES	SAM Large LM fine-tuned
	NO	Slow	YES	SAM Large EM fine-tuned
	NO	YES	YES	MobileSAM
	NO	YES	YES	MobileSAM LM fine-tuned
	NO	YES	YES	MobileSAM EM fine-tuned
QuPath–SAM	NO	NO	YES	SAM Huge LM fine-tuned
	NO	NO	YES	SAM Huge EM fine-tuned
	NO	Slow	YES	SAM Huge
	NO	Slow	YES	SAM Large
	NO	Slow	YES	SAM Base
	NO	YES	YES	MobileSAM
	NO	YES	YES	SAM2 Tiny
	NO	YES	YES	SAM2 Small
	NO	YES	YES	SAM2 Base Plus
	NO	YES	YES	SAM2 Large

*LM* Light Microscopy, *EM* Electron Microscopy.

**Table 3:** CPU-only model loading and annotation times across software tools

Software	Model	Load time (s)	Annotation time (s)
SAMJ	SAM2 Tiny	1.91	0.06
	SAM2 Small	2.00	0.07
	SAM2 Large	3.56	0.07
	EfficientSAM	2.69	0.08
	EfficientViTSAM-L2	1.37	0.08
Micro–SAM	MobileSAM LM	2.27	0.04
	MobileSAM EM	1.98	0.03
	SAM Base LM	8.65	0.05
	SAM Base EM	8.38	0.07
	SAM Large LM	26.03	0.06
	SAM Large EM	25.03	0.05
	SAM Huge	39.39	0.04
QuPathSAM	SAM2 Tiny	NA	0.31
	SAM2 Small LM	NA	0.32
	SAM2 Base Plus EM	NA	0.58
	MobileSAM LM	NA	0.25
	SAM Huge EM	NA	Only GPU
	SAM Huge LM	NA	Only GPU

Load time denotes the time required to load a model and encode the image on the CPU before annotation begins. For QuPathSAM, this metric is not applicable because the software does not pre-encode the image; instead, it encodes only the region around the user-provided prompt. If subsequent prompts fall within an already encoded region, no additional computation is required; otherwise, QuPathSAM performs a new local encoding step.

**Table 4:** IoU of annotations produced by each model across datasets

Software	Model	^21^	^22^	^23^	^24^	^25^	^25^	^26^	^27^
		C	C	B	C	N	C	M	M
SAMJ	SAM2 Tiny*	0.64	0.35	0.36	0.57	0.66	0.45	0.79	0.69
	SAM2 Tiny^†^	0.77	0.74	0.74	0.84	0.81	0.72	0.88	0.82
	SAM2 Small*	0.63	0.35	0.36	0.57	0.67	0.42	0.82	0.71
	SAM2 Small^†^	0.77	0.73	0.74	0.84	0.82	0.73	0.88	0.82
	SAM2 Large*	0.66	0.37	0.42	0.60	0.70	0.43	0.82	0.73
	SAM2 Large^†^	0.78	0.74	0.73	0.84	0.81	0.72	0.88	0.82
	EfficientSAM*	0.63	0.40	0.39	0.55	0.68	0.42	0.69	0.70
	EfficientSAM^†^	0.74	0.69	0.68	0.81	0.77	0.62	0.87	0.79
	EfficientViTSAM-L2*	0.69	0.50	0.41	0.61	0.71	0.47	0.82	0.73
	EfficientViTSAM-L2^†^	0.80	0.77	0.76	0.86	0.85	0.76	0.90	0.86
Micro-SAM	MobileSAM LM*	0.69	0.60	0.60	0.77	0.75	0.66	0.33	0.65
	MobileSAM LM^†^	0.82	0.80	0.76	0.85	0.84	0.80	0.77	0.84
	MobileSAM EM*	0.48	0.47	0.40	0.60	0.67	0.56	0.39	0.76
	MobileSAM EM^†^	0.62	0.71	0.62	0.78	0.87	0.77	0.79	0.87
	SAM Base LM*	0.74	0.69	0.70	0.82	0.74	0.70	0.46	0.69
	SAM Base LM^†^	0.85	0.83	0.82	0.89	0.84	0.82	0.79	0.84
	SAM Base EM*	0.58	0.49	0.38	0.58	0.64	0.53	0.53	0.81
	SAM Base EM^†^	0.77	0.71	0.65	0.76	0.84	0.78	0.80	0.88
	SAM Large LM*	0.77	0.71	0.74	0.83	0.75	0.71	0.49	0.69
	SAM Large LM^†^	0.86	0.83	0.83	0.89	0.84	0.83	0.78	0.84
	SAM Large EM*	0.57	0.50	0.41	0.57	0.62	0.53	0.59	0.82
	SAM Large EM^†^	0.76	0.69	0.63	0.75	0.84	0.78	0.78	0.89
	SAM Huge*	0.64	0.28	0.30	0.55	0.64	0.35	0.44	0.70
	SAM Huge^†^	0.76	0.69	0.62	0.75	0.79	0.59	0.71	0.82
QuPath–SAM	SAM2 Tiny*	0.57	0.29	0.32	0.57	0.28	0.36	0.41	0.75
	SAM2 Tiny^†^	0.74	0.71	0.65	0.78	0.72	0.62	0.65	0.84
	SAM2 Small*	0.56	0.32	0.32	0.55	0.25	0.34	0.42	0.70
	SAM2 Small^†^	0.76	0.68	0.64	0.77	0.75	0.65	0.65	0.84
	SAM2 Base+*	0.59	0.40	0.31	0.55	0.25	0.38	0.42	0.59
	SAM2 Base+^†^	0.75	0.70	0.63	0.76	0.71	0.64	0.65	0.84
	MobileSAM*	0.47	0.25	0.31	0.54	0.20	0.27	0.25	0.51
	MobileSAM^†^	0.71	0.62	0.61	0.73	0.68	0.57	0.64	0.77
	SAM Huge LM*	0.66	0.69	0.70	0.77	0.45	0.53	0.29	0.67
	SAM Huge LM^†^	0.75	0.80	0.81	0.87	0.68	0.64	0.69	0.73
	SAM Huge EM*	0.59	0.45	0.46	0.56	0.48	0.51	0.46	0.81
	SAM Huge EM^†^	0.74	0.65	0.70	0.72	0.63	0.68	0.71	0.80

For each software tool and model variant, we report the IoU (Intersection over Union) between the predicted masks and the ground-truth annotations on the following benchmark datasets: Cellpose^21^, LiveCell^22^, DeepBacs^23^, NeurIPS Cell Segmentation^24^, TissueNet Nuclei^25^, TissueNet Cells^25^, MitoEM^26^ and CEM-MitoLab^27^. Importantly, this evaluation measures *annotation performance*, not full-image segmentation: prompts were automatically derived from the ground-truth labels and each tool generated a corresponding mask, which we compared directly to the ground truth. For conciseness, in Micro-SAM and QuPath-SAM we evaluated only the fine-tuned SAM variants and SAM Huge. The fine-tuned models are expected to perform best on cellular data, and SAM Huge is not available in SAMJ.

*3-point multipoint prompt, ^†^bounding box prompt, *C* cells, *B* bacteria, *N* nuclei, *M* mitochondria, *LM* fine-tuned on light microscopy images, *EM* fine-tuned on electron microscopy images.

Importantly, our benchmarking targets annotation performance, not full image segmentation. Annotation requires the tool to correctly identify and delineate an object given a prompt–that is, the user (or, in our evaluation, the ground truth) indicates where the object is, and the tool produces the corresponding mask. To evaluate annotation accuracy, we generated prompts directly from the ground-truth labels, produced annotations in each software using those prompts, and then compared the resulting masks with the ground-truth masks.

As a final remark, we note that QuPath-SAM and Micro-SAM also provide access to the original SAM models trained on natural images. To keep the comparison focused and the tables concise, we restricted our evaluation to the fine-tuned variants, which are expected to perform more reliably on cellular datasets. We additionally include SAM Huge in our benchmarking, as this model is available on Micro-SAM but not in SAMJ.

### Enabling seamless integration of SAM with Fiji: the role of appose and SAMJ

**Fig. 4:**
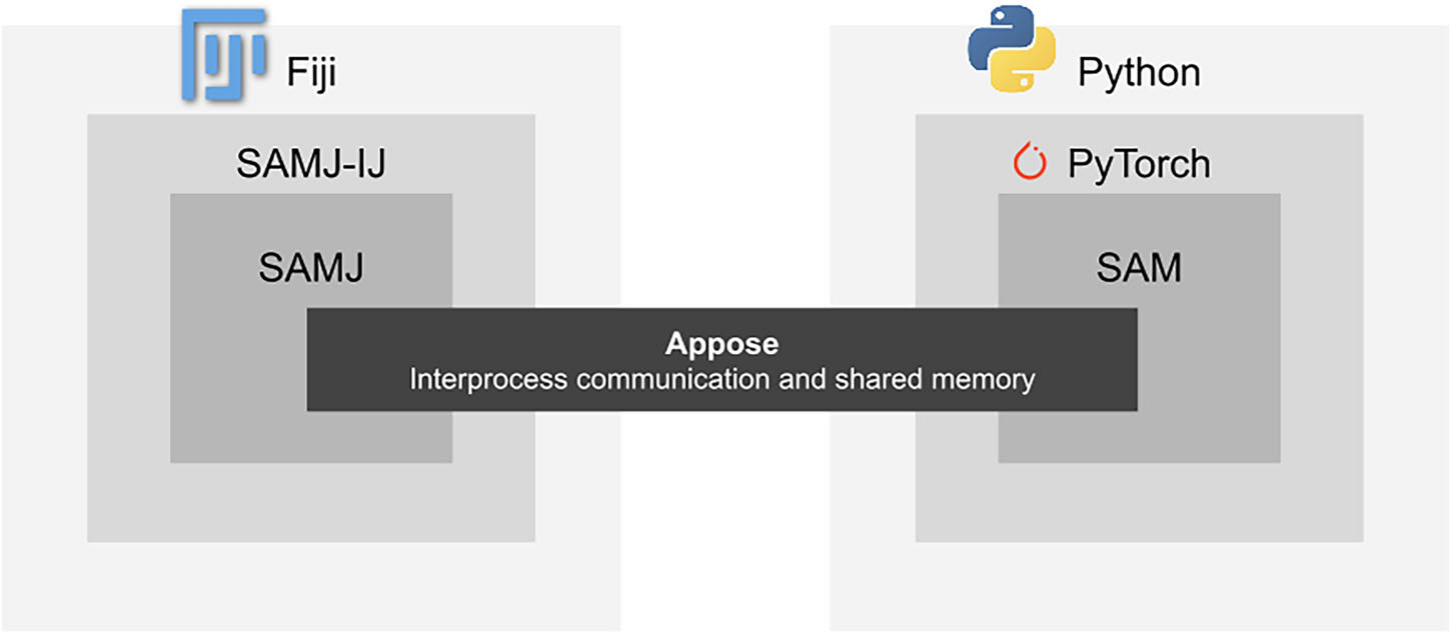
Overview of the integration between SAMJ and SAM via Appose. On the left, the Fiji/ImageJ environment hosts the SAMJ plugin, which acts as the interface for users within Java-based image processing tools. On the right, the Python with Pytorch runs the SAM model for segmentation tasks. Appose bridges these two environments, enabling seamless inter-process communication between Java and Python. It facilitates variable exchange via pipes and shared memory, ensuring efficient handling of images/tensors while avoiding memory duplication. This architecture encapsulates processes, preventing conflicts, and allows for the concurrent execution of multiple models.

The integration of SAM into the Fiji ecosystem via SAMJ is facilitated by Appose^16^ (see Fig. 4), a software framework designed to streamline inter-process communication. Within SAMJ, Appose orchestrates communication between the Java environment–where Fiji and SAMJ reside–and the Python environment, which runs SAM’s inference via PyTorch.

Appose employs process pipes to transmit variables between Java and Python and shared memory to exchange images and tensors efficiently, thus avoiding unnecessary data duplication. This architecture neatly segregates computational tasks, mitigating the risk of memory conflicts and enabling multiple models to be run concurrently without interference. Once a model is deselected, the associated processes are automatically terminated, ensuring a clean and self-contained execution environment.

In addition to managing communication, Appose automatically handles the installation and setup of required software environments. Using Micromamba, it can create and maintain isolated environments with all necessary dependencies without requiring user intervention. This fully automated process ensures a seamless user experience, simplifying the deployment and use of SAM within Fiji.

Appose creates a robust foundation for SAMJ to function as a powerful Java-to-Python bridge. This allows users to integrate SAM’s advanced segmentation capabilities, enabling the annotation of images in the Fiji ecosystem, benefiting from all tools, plugins, and capabilities of Java. Appose and SAMJ mark a significant checkpoint in the integration of novel, cutting-edge technologies developed in Python into the user-friendly world of Java.

### Reporting summary

Further information on research design is available in the Nature Portfolio Reporting Summary linked to this article.

## Supplementary information

Supplementary Information

Reporting Summary

Transparent Peer Review file

## Source data

Source Data

## Data Availability

The datasets used in this study are publicly available and are referenced throughout the manuscript. No new datasets were generated for this work. Source data are provided with this paper.

## Code availability

The complete source code for the SAMJ platform, encompassing the library’s core Java implementation, its ImageJ/Fiji integration, and associated components, is accessible at https://github.com/segment-anything-models-java. For comprehensive documentation, usage instructions, and examples, please refer to the SAMJ-IJ README available at https://github.com/segment-anything-models-java/SAMJ-IJ/blob/main/README.md.

## References

[1] Pelt, D. M. Tackling the challenges of bioimage analysis. *Elife* **9**, e64384 (2020). 33264089 10.7554/eLife.64384 PMC7710355

[2] Zulueta-Coarasa, T. et al. Mifa: Metadata, Incentives, Formats, and Accessibility guidelines to improve the reuse of AI datasets for bioimage analysis. Nat. Methods **22**, 2245–2252 (2025). 10.1038/s41592-025-02835-8 40954297

[3] Schindelin, J. et al. Fiji: an open-source platform for biological-image analysis. *Nat. Methods* **9**, 676–682 (2012). 22743772 10.1038/nmeth.2019 PMC3855844

[4] Kirillov, A. et al. Segment anything. In *Proceedings of the IEEE/CVF International Conference on Computer Vision*, 4015–4026 (2023).

[5] Sugawara, K. Training deep learning models for cell image segmentation with sparse annotations. *BioRxiv* 2023–06 (2023).

[6] Archit, A. et al. Segment anything for microscopy. *Nat. Methods* **22**, 579–591 (2025). 10.1038/s41592-024-02580-4 PMC11903314 39939717

[7] Schindelin, J., Rueden, C. T., Hiner, M. C. & Eliceiri, K. W. The ImageJ ecosystem: An open platform for biomedical image analysis. *Mol. Reprod. Dev.* **82**, 518–529 (2015). 26153368 10.1002/mrd.22489 PMC5428984

[8] Hollandi, R., Diósdi, Á, Hollandi, G., Moshkov, N. & Horváth, P. Annotatorj: an imageJ plugin to ease hand annotation of cellular compartments. *Mol. Biol. cell* **31**, 2179–2186 (2020). 32697683 10.1091/mbc.E20-02-0156 PMC7550707

[9] Arzt, M. et al. Labkit: labeling and segmentation toolkit for big image data. *Front. Computer Sci.* **4**, 777728 (2022).

[10] Thomas, L. S., Schaefer, F. & Gehrig, J. Fiji plugins for qualitative image annotations: routine analysis and application to image classification. *F1000Res.* **9**, 1248 (2021). 10.12688/f1000research.26872.1 PMC8014705 33841801

[11] Dosovitskiy, A. et al. An image is worth 16x16 words: Transformers for image recognition at scale. In *International Conference on Learning Representations*. https://openreview.net/forum?id=YicbFdNTTy (2021).

[12] Ravi, N. et al. Sam 2: Segment anything in images and videos. arXiv preprint arXiv:2408.00714 (2024).

[13] Xiong, Y. et al. Efficientsam: Leveraged masked image pretraining for efficient segment anything. In *Proceedings of the IEEE/CVF Conference on Computer Vision and Pattern Recognition*, 16111–16121 (2024).

[14] Zhang, Z., Cai, H. & Han, S. Efficientvit-sam: Accelerated segment anything model without performance loss. In *Proceedings of the IEEE/CVF Conference on Computer Vision and Pattern Recognition*, 7859–7863 (2024).

[15] Pietzsch, T., Saalfeld, S., Preibisch, S. & Tomancak, P. Bigdataviewer: visualization and processing for large image data sets. *Nat. Methods* **12**, 481–483 (2015). 26020499 10.1038/nmeth.3392

[16] Appose Developers. Appose: Run Python from Java using separate processes. GitHub repository, accessed 2026-03-04. https://github.com/apposed/appose (2024).

[17] QuantStack and Mamba Contributors. Mamba: A fast, robust, and cross-platform package manager for the conda ecosystem https://mamba.readthedocs.io/ (2019).

[18] Schmidt, U., Weigert, M., Broaddus, C. & Myers, G. Cell detection with star-convex polygons. In *Medical image computing and computer assisted intervention–MICCAI 2018: 21st international conference, Granada, Spain, September 16-20, 2018, proceedings, part II 11*, 265–273 (Springer, 2018).

[19] Gómez-de Mariscal, E. et al. DeepImageJ: A user-friendly environment to run deep learning models in Image. *J. Nat. Methods* **18**, 1192–1195 (2021). 10.1038/s41592-021-01262-9 34594030

[20] Casado-Garcia, A. et al. Motilityj: An open-source tool for the classification and segmentation of bacteria on motility images. *Computers Biol. Med.* **136**, 104673 (2021). 10.1016/j.compbiomed.2021.104673 34325228

[21] Stringer, C., Wang, T., Michaelos, M. & Pachitariu, M. Cellpose: a generalist algorithm for cellular segmentation. *Nat. Methods* **18**, 100–106 (2021). 33318659 10.1038/s41592-020-01018-x

[22] Edlund, C. et al. Livecell-a large-scale dataset for label-free live cell segmentation. *Nat. Methods* **18**, 1038–1045 (2021). 34462594 10.1038/s41592-021-01249-6 PMC8440198

[23] Spahn, C. et al. Deepbacs for multi-task bacterial image analysis using open-source deep learning approaches. *Commun. Biol.* **5**, 688 (2022). 35810255 10.1038/s42003-022-03634-z PMC9271087

[24] Ma, J. et al. The multi-modality cell segmentation challenge: Towards universal solutions. *Nat. Methods* **21**, 1103–1113 (2024). 38532015 10.1038/s41592-024-02233-6 PMC11210294

[25] Greenwald, N. F. et al. Whole-cell segmentation of tissue images with human-level performance using large-scale data annotation and deep learning. *Nat. Biotechnol.* **40**, 555–565 (2022). 34795433 10.1038/s41587-021-01094-0 PMC9010346

[26] Wei, D. et al. Mitoem dataset: Large-scale 3d mitochondria instance segmentation from em images. In *International Conference on Medical Image Computing and Computer-Assisted Intervention*, 66–76 (Springer, 2020). 10.1007/978-3-030-59722-1_7 PMC7713709 33283212

[27] Conrad, R. & Narayan, K. Instance segmentation of mitochondria in electron microscopy images with a generalist deep learning model trained on a diverse dataset. *Cell Syst.* **14**, 58–71 (2023). 36657391 10.1016/j.cels.2022.12.006 PMC9883049

[28] Shamai, G. et al. Deep learning-based image analysis predicts PD-L1 status from H&E-stained histopathology images in breast cancer. *Nat. Commun.* **13**, 6753 (2022). 36347854 10.1038/s41467-022-34275-9 PMC9643479

[29] Casser, V., Kang, K., Pfister, H. & Haehn, D. Fast mitochondria detection for connectomics. In *Medical Imaging with Deep Learning*, 111–120 (PMLR, 2020).

[30] Morelli, R. et al. Automating cell counting in fluorescent microscopy through deep learning with c-ResUnet. *Sci. Rep.* **11**, 22920 (2021). 34824294 10.1038/s41598-021-01929-5 PMC8617067

