# Supplementary material for: SAMJ: fast image annotation on ImageJ/Fiji via segment anything model
Source: Nat Commun. 2026 Jun 18;17:5402. doi: 10.1038/s41467-026-71752-x (PMC13279790; doi:10.1038/s41467-026-71752-x)
Supplement: Supplementary file 1 — Supplementary Information [file 41467_2026_71752_MOESM1_ESM.pdf]

Supplementary Information

This document contains supplementary material for our manuscript “SAMJ: fast image annotation on ImageJ/Fiji via Segment Anything Model”. It contains more extended descriptions of our software implementation and our user studies that did not fit into the methods section. In addition, it links supplementary videos that contain tutorials for each of the use cases.

1 Available SAM Variants

The original SAM model is computationally intensive and was initially introduced in three versions, differentiated by the size of the Vision Transformer (ViT): base, large, and huge. However, even the smallest model, SAM-base, is too heavy to run on standard CPUs due to its high resource demands.

To address these limitations, newer and more lightweight variants have been developed. These models (efficientSAM [1]; efficientViTSAM [2]) achieve a significant improvement in computational efficiency with only a minimal trade-off in annotation quality, making SAM-like models accessible for lower-end CPUs.

Moreover, the release of the second generation of the original SAM, known as SAM-2 [3], marked a breakthrough. This new family of models not only enhances annotation quality but also significantly reduces computational costs.

For these reasons, the list of models available in SAMJ does not include any of the original SAM versions. This list is expected to evolve as new models achieve improvements in performance and efficiency. Currently, the supported models in SAMJ include: **SAM-2 (tiny, small, and large)**, **EfficientSAM**, and **EfficientViTSAM** (see Table 1). For the most up-to-date list, please refer to the official SAMJ GitHub repository: https://github.com/segment-anything-models-java/SAMJ/blob/main/README.md.

	**Model Size (MB)**	**Time to encode (seconds)**	**In SAMJ**
SAM-2 Tiny	148.7	1.14	Yes
SAM-2 Small	175.8	1.39	Yes
SAM-2 Large	856.4	5.59	Yes
EfficientSAM	105.7	3.19	Yes
EfficientViTSAM-l2	245.7	0.74	Yes
SAM Base	375.0	15	No
SAM Large	1191.64	15	No
SAM Huge	2445.74	15	No

Supplementary Table 1: **Encoding time comparison for SAM-2, EfficientSAM, and original SAM models.** The table shows model sizes (in MB) and the time required (in seconds) to perform encoding. Lightweight models such as SAM-2 Tiny and EfficientViTSAM-l2 significantly reduce both size and encoding time compared to the original SAM models. All results in this table were obtained on a workstation equipped with a 13th Gen Intel® Core™ i7-13700H processor and 32 GB of RAM. Although running the model on different hardware may produce varying absolute timings, the relative performance differences are expected to remain consistent.

2 SAMJ Embedding Strategy

To generate annotations, SAM must first embed the image, or at least a portion of it. This step creates an embedding of the image, which is then used to generate subsequent annotations. The

	500x500	1000x1000	2000x2000	3000x3000	4000x4000
Object: 26x26	0.808	**0.828**	**0.828**	**0.828**	**0.828**
Object: 52x52	0.927	0.930	0.930	0.930	0.930
Object: 78x78	**0.956**	0.944	0.933	0.933	0.933
Object: 104x104	**0.953**	**0.959**	0.929	0.929	0.929
Object: 130x130	**0.968**	**0.958**	0.932	0.920	0.920
Object: 156x156	**0.967**	**0.969**	0.927	0.903	0.903
Object: 182x182	**0.969**	**0.974**	0.936	0.912	0.873
Object: 208x208	**0.969**	**0.977**	0.929	0.892	0.871
Object: 234x234	**0.975**	**0.970**	**0.957**	0.921	0.868
Object: 260x260	0.611	**0.976**	0.946	0.898	0.879
Object: 286x286	0.612	**0.978**	0.940	0.911	0.898
Object: 312x312	0.542	**0.972**	**0.954**	0.907	0.888
Object: 338x338	0.429	**0.976**	**0.951**	0.922	0.910
Object: 364x364	0.566	**0.969**	**0.965**	**0.979**	0.899
Object: 390x390	**0.974**	**0.979**	**0.976**	0.919	0.913
Object: 416x416	0.000	**0.976**	**0.974**	0.903	0.891
Object: 442x442	0.459	**0.983**	**0.976**	0.931	0.907

Supplementary Table 2: Impact of object size and image resolution on SAM detection scores. The table shows detection scores for objects of varying sizes across different image resolutions (from 500×500 to 4000×4000 pixels). Bold values indicate the maximum score in a row or scores ≥ 0.95. Results demonstrate that SAM performs optimally when objects maintain a balanced size relative to the image resolution, with sizes closer to 1024×1024 yielding higher and more consistent scores.

region for which the embedding has been computed determines the area SAMJ can work with to create annotations. If the user navigates to a region of the image that has not yet been processed, the embedding will need to be recalculated for that specific region before new annotations can be generated.

Having a clear strategy to define the portion of the image for which the embedding is created is therefore critical. In SAMJ, this is achieved by leveraging the interactive features provided by Fiji, such as the region of the image currently being viewed and user input. Depending on the user's input, the method for determining the portion of the image to be embedded varies (see Figure 1). This strategy aims to enhance the quality of annotations produced by SAMJ and enables annotations to be created for a wider range of object shapes and sizes.

Consequently, there may be slight differences between annotations generated by SAMJ and those produced using the original SAM implementation. These differences reflect the flexibility of SAMJ's embedding strategy in accommodating diverse annotation requirements.

3 Bounding Box Size vs. Image Size

SAM and its variants were primarily trained on natural images rescaled to a 1024×1024 pixel size. Under these training conditions, objects generally maintain a consistent size ratio relative to the image, which may bias the model towards favoring certain object-to-image size ratios at inference time.

As shown in Table 2, images with sizes closer to 1024×1024 pixels are better understood by SAM. Since SAM only accepts images at this fixed size, the more the input image must be rescaled, the more its quality and interpretability may degrade.

Additionally, the table suggests that objects should not be extremely large or very small to be correctly annotated. Finding a balanced object-to-image size ratio appears crucial for achieving optimal performance.

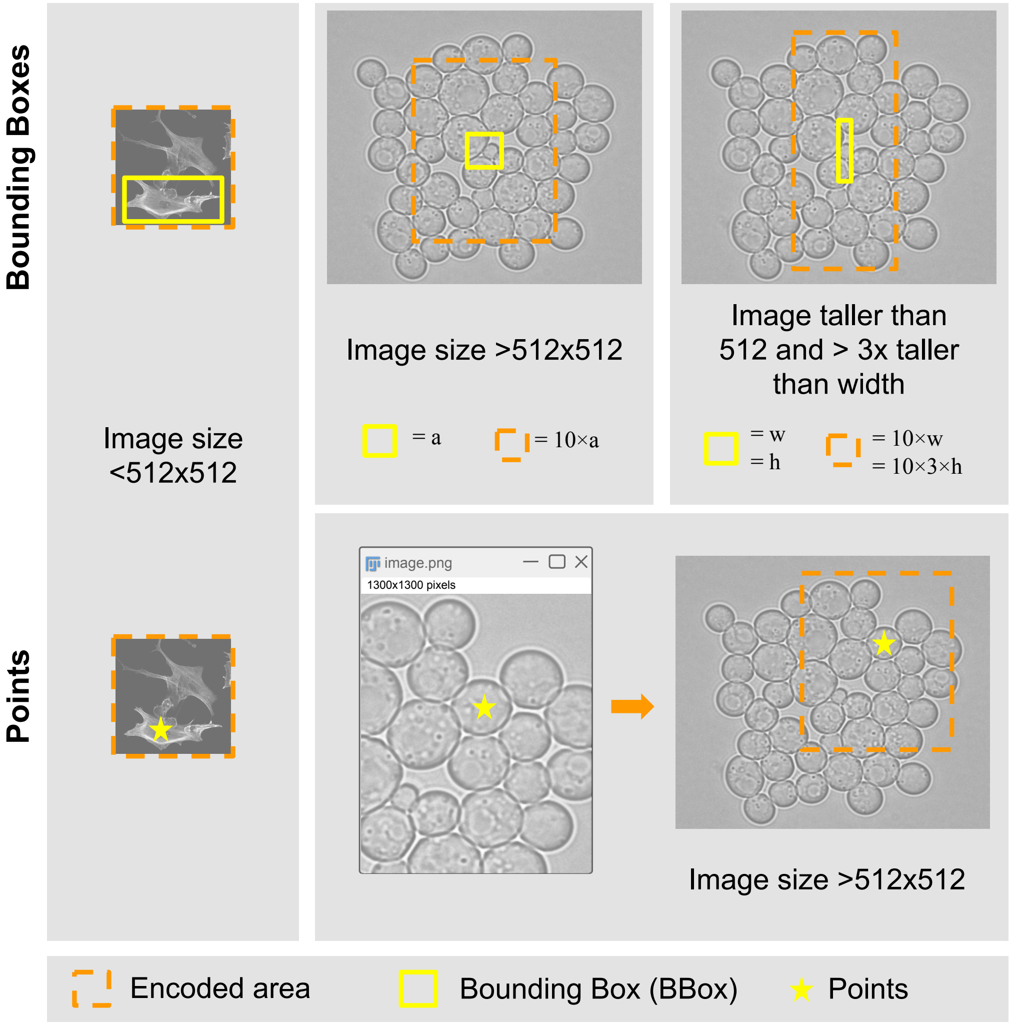

Supplementary Figure 1: **Embedding strategies.** SAMJ generates embeddings for regions of the image based on the user-defined input and the size of the image. For smaller images (smaller than 512 × 512 pixels), the entire image is embedded regardless of the prompt. For larger images (bigger than 512 × 512 pixels), different strategies are followed depending on the prompt and size of the bounding box. If the bounding box is relatively regular, the embedded area will be ten times the size of the bounding box. If one side of the bounding box is three times smaller than the other side of the bounding box, the embedded area will extend ten times the length of the smaller side and ten-thirds (approximately 3.33 times) the length of the larger side. For larger images and point prompts, the embedded area will include the visible region in the Fiji window plus an additional margin.

4 Extending SAMJ for Large-Scale and Multi-dimensional Image Annotation with BigDataViewer

Building on the SAMJ plugin, we developed an extension for BigDataViewer, a core Fiji tool for visualizing large and multi-dimensional images [4]. This extension enables users to annotate these types of images by drawing rectangular prompts directly on the displayed image, which are immediately processed by the selected SAM model from the SAMJ Annotation control panel. The resulting polygons are rendered over the image and stored with their spatio-temporal coordinates. Polygons are displayed only when the user navigates back to the corresponding view angle and time point, but to simplify navigation, the extension memorizes view configurations, allowing users to cycle through and revisit them seamlessly. New view configurations trigger automatic image embedding, and additional features such as an undo/redo mechanism and mask export to Fiji further enhance usability.

The software is a lightweight library designed to integrate with an existing BigDataViewer instance, enabling users to add prompts directly. It allows client programs to define custom routines triggered when prompts are entered or new polygons are created. Our implementation facilitates communication between BigDataViewer and SAM models using this mechanism, while also enabling client programs to override default behaviors—for instance, converting prompts into polygons or customizing how polygons are rendered. This flexibility has been applied in Mastodon (a large-scale tracking framework for large, multi-view images), where polygons are replaced with spots for tracking, and in Labkit, where SAM-generated polygons are used for pixel classification labels.

This new library for BigDataViewer is central to enabling efficient annotation of large and multi-dimensional images. By connecting SAMJ with BigDataViewer, users can seamlessly annotate much larger datasets, including those in 3D, significantly expanding the scope of bioimage analysis.

5 Usability and troubleshooting

SAMJ was designed with accessibility and usability in mind, but we recognize that new users may still face challenges. To facilitate onboarding, we provide a set of short demo videos in subsection 7, which offer a quick start for most use cases.

Because SAM is a computationally demanding model, we incorporated several SAM variants so that users can select an option suitable for their hardware. On computers with limited resources, loading a large model may take several minutes, which can be mistaken for a crash. In such cases we recommend starting with lighter variants (e.g., EfficientViT-L2 or EfficientSAM) and then moving to larger models if performance allows. For reference, all development was carried out on a Linux workstation with an Intel i7 CPU and 32 GB RAM, where models typically load in under 6 seconds. Even on an older 10-year-old Intel i3 with 16 GB RAM, SAM2 Small loaded in under two minutes.

Users should also note that the first model load and the first annotation after installation or system startup are slower than subsequent runs, since Python needs to cache required libraries. For example, the first load of SAM2 Tiny may take 1–2 minutes and the first annotation up to 7–9 seconds, while subsequent operations are nearly instantaneous.

Each model is installed in its own Python environment, stored in the `Appose` directory under the Fiji/ImageJ folder. Although these environments can be accessed directly in Python, we recommend not modifying them, as changes may break SAMJ's configuration. If an environment becomes inconsistent (e.g., missing libraries or version mismatches), SAMJ will prompt reinstallation, which replaces the old environment, deleting the user's work if they had done any there.

Finally, for troubleshooting or suggestions, users are encouraged to post questions on the Image.sc forum or to open an issue on GitHub (https://github.com/segment-anything-models-java/SAMJ-IJ/issues).

6 Example SAMJ macros

SAMJ supports macro usage via BatchSAMize with preset prompts. Here we show three examples demonstrating how to integrate SAMJ into ImageJ workflows using macros.

Single-prompt example

The following macro creates a single-point prompt in ImageJ and annotates it with SAMJ.

```
// Open example image
run("Blobs (25K)");

// USe the "multipoint" the tool and create a prompt
makePoint(177, 33, "small yellow hybrid");

// Use SAM2 Tiny to annotate the selected object.
// This command also generates a label mask.
run("SAMJ Annotator", "model=[SAM2 Tiny] export=true");
```

Automatic prompts using Find Maxima

This macro uses ImageJ's Find Maxima command to automatically generate prompts for all detected objects. SAMJ then annotates each instance.

```
// Open example image
run("Blobs (25K)");

// Invert the image so objects are white and the background is black
run("Invert");

// Use ImageJ Find Maxima to detect one prompt per instance
run("Find Maxima...", "prominence=100 output=[Point Selection]");

// Use SAM2 Tiny to annotate all detected prompts.
// This command also generates a label mask.
run("SAMJ Annotator", "model=[SAM2 Tiny] export=true");
```

Processing all images in a folder

This macro processes every image inside a folder. For each image, it detects instance-level prompts via Find Maxima and annotates them with SAMJ.

```
// Set the directory that contains all the images you want to annotate
directory = "/path/to/images/folder";
files = getFileList(directory);

for (i = 0; i < files.length; i++) {
    // Open each of the images of the folder
    open(directory + File.separator + files[i]);
    selectImage(files[i]);

    // Use ImageJ Find Maxima to detect one prompt per instance
    run("Find Maxima...", "prominence=100 output=[Point Selection]");

```

```
 // Use SAM2 Tiny to annotate all detected prompts.
 // This command also generates a label mask.
 run("SAMJ Annotator", "model=[SAM2 Tiny] export=true");
}
```

7 Supplementary videos

- SAMJ: Nuclei Segmentation using Fiji's capabilities and BatchSAMize: https://www.youtube.com/watch?v=vp0zqLyxzrk
- SAMJ: Tumor Area Quantification and Nuclei Analysis with SAMJ and StarDist plugins for Fiji: https://www.youtube.com/watch?v=4JhSEtDxY9g
- SAMJ: Accelerating Annotation of Bacterial Motility: https://www.youtube.com/watch?v=10PTr5DkgBc
- SAMJ: Integration with Labkit: https://www.youtube.com/watch?v=2l0cyjNm8Oo

References

[1] Xiong, Y., Varadarajan, B., Wu, L., Xiang, X., Xiao, F., Zhu, C., Dai, X., Wang, D., Sun, F., Iandola, F., *et al.*: Efficientsam: Leveraged masked image pretraining for efficient segment anything. In: Proceedings of the IEEE/CVF Conference on Computer Vision and Pattern Recognition, pp. 16111–16121 (2024)

[2] Zhang, Z., Cai, H., Han, S.: Efficientvit-sam: Accelerated segment anything model without performance loss. In: Proceedings of the IEEE/CVF Conference on Computer Vision and Pattern Recognition, pp. 7859–7863 (2024)

[3] Ravi, N., Gabeur, V., Hu, Y.-T., Hu, R., Ryali, C., Ma, T., Khedr, H., Rädle, R., Rolland, C., Gustafson, L., et al.: Sam 2: Segment anything in images and videos. arXiv preprint arXiv:2408.00714 (2024)

[4] Pietzsch, T., Saalfeld, S., Preibisch, S., Tomancak, P.: Bigdataviewer: visualization and processing for large image data sets. Nature methods **12**(6), 481–483 (2015)
