# Supplementary material for: SAMJ: fast image annotation on ImageJ/Fiji via segment anything model
Source: Nat Commun. 2026 Jun 18;17:5402. doi: 10.1038/s41467-026-71752-x (PMC13279790; doi:10.1038/s41467-026-71752-x)
Supplement: Supplementary file 3 — Transparent Peer Review file [file 41467_2026_71752_MOESM3_ESM.pdf]

nature portfolio

Peer Review File

SAMJ: fast image annotation on ImageJ/Fiji via Segment Anything Model

Corresponding Author: Professor Arrate Muñoz-Barrutia

This file contains all reviewer reports in order by version, followed by all author rebuttals in order by version.

Version 0:

Reviewer comments:

Reviewer #1

(Remarks to the Author)
Manuscript Background Information
Title: SAMJ: Fast Image Annotation on ImageJ/Fiji via Segment Anything Model
Authors: Carlos García-López-de-Haro, Caterina Fuster-Barceló, Curtis T. Rueden, Jónathan Heras, Vladimír Ulman, et al.
Summary:
This manuscript introduces SAMJ, an ImageJ/Fiji plugin that integrates the capabilities of the Segment Anything Model (SAM) into the widely used Fiji ecosystem. SAMJ offers a user-friendly, no-code interface, enabling one-click installation and efficient image annotation, particularly for biomedical image analysis. The tool emphasizes compatibility with standard hardware, accessibility for non-technical users, and seamless integration with existing Fiji tools such as Labkit and BigDataViewer.
The authors showcase SAMJ's effectiveness through four use cases:
• Nuclei segmentation using Fiji and BatchSAMize
• Tumor area and nuclei quantification in cancer histopathology images
• Bacterial motility annotation
• 3D mitochondria annotation in EM volumes
SAMJ's innovation lies in bridging Python-powered models like SAM with Java-based platforms using Appose. It simplifies model installation via Micromamba and allows annotation via bounding boxes or point prompts. Performance benchmarks and embedding strategies are also detailed.
Significance:
SAMJ addresses a significant bottleneck in biomedical deep learning rapid and scalable annotation. By integrating SAM into Fiji and enabling efficient use on mid-range machines, it democratizes advanced segmentation tools for broader scientific audiences.

(Remarks on code availability)
Dear Authors,
Thank you for submitting your manuscript titled "SAMJ: Fast Image Annotation on ImageJ/Fiji via Segment Anything Model." The manuscript presents an important and practical advancement for the bioimage analysis community. Your efforts to bring cutting-edge segmentation tools into Fiji through a user-friendly plugin are commendable.
Strengths:
• The design of SAMJ is accessible, efficient, and versatile across a range of biomedical use cases.
• Integration with Fiji, Labkit, and BigDataViewer positions SAMJ well within existing workflows.
• One-click installation and support for mid-range hardware significantly lower the entry barrier for non-expert users.
• The presented use cases are clear, relevant, and effectively demonstrate the plugin's power.
Suggestions for Improvement:
1. Quantitative Evaluation: While visual examples are informative, the addition of standard performance metrics (e.g., IoU, Dice score) across the use cases would provide stronger validation.
2. User Feedback: Consider including results or a short discussion from a preliminary user survey or usability evaluation, particularly with non-technical biologists.
3. Documentation & Stability: Please comment briefly on the long-term maintenance plans, especially for keeping compatibility with evolving versions of SAM and Fiji.

4. Batch Mode Flexibility: While BatchSAMize is a valuable feature, clarifying its extensibility to more complex prompt types or segmentation goals could enhance reproducibility.
Overall, this is a well-structured and impactful contribution. With minor revisions, it is well-suited for publication.

Reviewer #2

(Remarks to the Author)
The manuscript under review: “SAMJ: Fast Image Annotation on ImageJ/Fiji via Segment Anything Model” describes a novel integration of the Segment Anything Model (SAM) with bioimaging-compatible tools such as ImageJ/Fiji to improve automatic annotation for biomedical image analysis and object segmentation. The authors describe SAMJ as an easy-to-use, accessible plugin that brings SAM capabilities, a deep learning Meta AI framework with promptable and one-click image segmentation, into the ImageJ ecosystem. The SAMJ workflow is outlined, and several examples were given to illustrate the application of SAMJ in segmenting a range of biological sample types, including nuclei, tumor area, bacteria motility, and 3D mitochondrial annotation from EM images.

General comments:
The article is generally well-written and provides a concise overview of the potential applications for SAMJ. Harnessing SAM for streamlining image annotation and segmentation, especially for complex structures found in cells and tissue has important implications for the field of biomedical science. The authors make several claims about the advantages of SAMJ, mostly relating to the acceleration and precision (page 7, lines 190-191) of user-driven annotation. Additionally, several other AI-assisted tools and plugins were mentioned throughout the manuscript. This study would greatly benefit by substantiating these claims through conducting a more systematic evaluation (e.g. speed, accuracy, robustness metrics) of SAMJ compared to other available tools. It would be helpful for the reader to understand how SAMJ objectively performs, how it offers tangible benefits to the end-user, and its limitations.

Specific comments:
1. The authors mention that “SAM has been made available in commonly used bioimage software packages” (page 2, line 58). Has SAM been integrated with other tools already and if so, how is SAMJ differentiated from them? For example, there appears to be other SAM adaptations that have been described in the field, such as ‘MedSAM’ (Ma et al., 2024).
2. Page 4, lines 97-100: the article states that “five SAM variants are available through SAMJ”, yet only three are mentioned in the paragraph. What are these variants and what do they do?
3. It is described that SAMJ offers two annotation modes – Live and Batch (page 2, line 108). In Batch mode, can you define seed points across different images? Can BatchSAMize be used to batch annotate a set of images?
4. Figure 2 highlights specific use cases to demonstrate the versatility of SAMJ applications. See below for questions regarding specific applications:
a. Can the authors demonstrate the use of SAMJ for segmenting subcellular structures (i.e. organelles) or cells with more complex morphologies (i.e. neurons)? The authors mention in page 6, line 135 that “elongated or branched structures may pose more of a challenge”. Can you speculate why this is? Is this an inherent limitation of the SAM foundational DL model?
b. In addition to tumor tissue, can SAMJ be used to automatically annotate complex tissue, such as brain regions?
c. The authors state that 3D segmentation is challenging in general. Can more details be shared on why SAMJ was successful in the mitochondrial use-case? How many slides does the user need to annotate for SAMJ to reliably segment mitochondrial within the entire tissue volume in 3D?
5. From a user-accessibility standpoint, can the authors provide further commentary on troubleshooting tips or suggestions to address most common hurdles when using SAMJ?
6. Relevant information regarding model comparison, performance and evaluation are found within the Appendix section. It would have been helpful to explicitly reference these supplementary tables and figures from the main text. Addressing this issue may in part, answer some of my comments above.

Overall evaluation:
Overall, the manuscript presents a valuable contribution to the field of bioimage analysis. SAMJ leverages automated and AI-assisted framework to potentially improve user-accessibility, annotation speed and precision. With the revisions detailed above, I would recommend this manuscript for publication in Nature Communications.

(Remarks on code availability)
The code is robust and the instructions provided were clear for installation. I was able to successfully run an example image on SAMJ to generate automated annotations within Fiji. The provided YouTube videos were also helpful as a step-by-step guide.

Version 1:

Reviewer comments:

Reviewer #1

(Remarks to the Author)
Comments for the Authors
The revised manuscript presents SAMJ, an ImageJ/Fiji plugin integrating the Segment Anything Model (SAM) and its

efficient variants into the Fiji ecosystem to facilitate fast, interactive image annotation. The authors have carefully addressed the main concerns raised in the previous review round, and the manuscript has improved significantly in terms of clarity, technical transparency, and scope definition
Below are specific comments intended to further strengthen the manuscript.
1. Clarity of Scope and Contribution (Well Addressed)
The authors now clearly state that SAMJ does not introduce a new segmentation model, but rather provides a software tool and workflow that enables the practical use of existing SAM models within Fiji. This clarification is important and effectively resolves earlier ambiguity regarding novelty. The distinction between annotation performance and full-image segmentation is now well articulated, particularly in the benchmarking sections and Appendix A.2.
2. Benchmarking and Comparative Evaluation (Improved)
The comparative analysis against Micro-SAM and QuPath-SAM is well designed and fair. The focus on:
• prompt-based annotation accuracy,
• CPU-only feasibility,
• model loading time and responsiveness,
is appropriate for the stated application domain. Tables A.2–A.4 provide valuable and practically relevant information for end users. The methodology for generating prompts from ground truth is clearly explained and reproducible.
Minor suggestion:
In a few places, qualitative statements such as "visually indistinguishable from ground truth" (e.g., bacterial motility use case) could be slightly strengthened by explicitly referencing the corresponding quantitative results already reported (e.g., IoU values in Table A.4), or by explicitly stating that these observations are qualitative.
3. Engineering and Integration Aspects (Strong)
The description of the Java–Python integration via Appose and Micromamba is clear, technically sound, and well documented. The automated environment handling and one-click installation represent a substantial usability improvement over existing SAM-based tools. Figures A.1 and A.2 effectively illustrate the embedding strategy and system architecture.
✔ This section is a clear strength of the manuscript.
4. Use Cases and Practical Demonstration (Well Executed)
The four use cases cover a broad range of imaging modalities (fluorescence, histopathology, bacterial motility, and 3D EM), convincingly demonstrating the versatility of SAMJ. The explanations are clearer than in the previous version, and the integration with Fiji tools such as Labkit, BigDataViewer, and macros is well motivated.
Minor suggestion:
A brief explicit mention of limitations in the main text (not only in the appendices) for example, challenges with highly elongated or extremely thin structures, or dependence on prompt quality would further improve transparency.
5. Reproducibility and Accessibility
The manuscript provides sufficient details regarding:
• hardware specifications,
• model variants,
• installation behavior,
• and expected performance on different systems.
The inclusion of example macros and tutorial videos further supports reproducibility and user adoption.
Summary
The revised manuscript is technically sound, clearly written, and practically valuable for the bioimage analysis community. The authors have responded thoughtfully to prior feedback, and only minor clarifications and wording refinements are suggested at this stage.

(Remarks on code availability)

Reviewer #2

(Remarks to the Author)
I thank the authors for carefully addressing my comments in full and for revising their manuscript text, figures, and methods to reflect the suggested changes. I recommend this revised manuscript for publication.

(Remarks on code availability)

Open Access This Peer Review File is licensed under a Creative Commons Attribution 4.0 International License, which permits use, sharing, adaptation, distribution and reproduction in any medium or format, as long as you give appropriate credit to the original author(s) and the source, provide a link to the Creative Commons license, and indicate if changes were made.
In cases where reviewers are anonymous, credit should be given to 'Anonymous Referee' and the source.
The images or other third party material in this Peer Review File are included in the article's Creative Commons license, unless indicated otherwise in a credit line to the material. If material is not included in the article's Creative Commons license and your intended use is not permitted by statutory regulation or exceeds the permitted use, you will need to obtain permission directly from the copyright holder.
To view a copy of this license, visit https://creativecommons.org/licenses/by/4.0/

Dear Editor and Reviewers,

We thank you for the thoughtful and constructive feedback on our manuscript, *"SAMJ: Fast Image Annotation on ImageJ/Fiji via Segment Anything Model"*. We are pleased that the assessment amounts to minor revisions, and we have revised the manuscript accordingly. Below, we provide a point-by-point response, indicating the exact changes made. Page and line references in reviewer comments are preserved as originally provided; in our responses, we refer to sections/figures/tables in the revised version for clarity.

We believe the revisions have substantially improved the clarity, scope, and reproducibility of the work while keeping the paper focused on SAMJ's primary contribution: a robust and user-friendly integration of Segment Anything Model (SAM) technology into the Fiji/ImageJ platform. See Fig. 1 for the end-to-end workflow timing and the installation/encoding/annotation breakdown; see Appendix A.1–A.9 for implementation details and comparisons.

Reviewer 1's comments:

First and foremost, we thank Reviewer 1 for their constructive and insightful comments. Addressing their suggestions has helped us clarify the scope, usability, and long-term vision of SAMJ. We appreciate the reviewer's careful reading and the opportunity their suggestions provided to strengthen both the clarity and practical value of the manuscript. We detail the changes made for each of Reviewer 1's comments in the points below.

Comment 1.1. Quantitative Evaluation: While visual examples are informative, the addition of standard performance metrics (e.g., IoU, Dice score) across the use cases would provide stronger validation.

Author answer:

We appreciate the reviewer's observation regarding quantitative benchmarking. SAMJ is conceived as an integration and workflow tool rather than a novel segmentation model; benchmarking model accuracy *'per se'* is outside its scope. To avoid model benchmarking with interface/workflow contribution, we have focused the main text on **accessibility and usability,** and now make these emphases even more explicit. Specifically, we now clarify in the manuscript that SAMJ does **not** aim to introduce a new SOTA segmenter, but rather to integrate SAM-family models inside Fiji for high-throughput annotation and macro/script automation.
However, we also acknowledge that SAM had not yet been benchmarked on microscopy images and thus it would be a useful guide for most users to understand how well SAM performs on different types of images and datasets. This is why we have now created a new section (*Online Methods Section A.2: Review of SAM-Based Annotation Tools for Bioimaging*) where we measure the annotation performance of the models supported by SAMJ (in addition to the models available in other SAM-based software) across several microscopy datasets.

Author changes, line 86:

“It is important to note that SAMJ does not introduce a novel model for bioimage segmentation. Instead, it provides a software tool that facilitates the use of an existing foundational model (SAM), which we believe can be highly valuable for bioimage annotation, especially within Fiji, where its environment, usability, and macro language make it an ideal setting for high-throughput annotation and automation.”

Author changes, added section in Online Methods, line 261:

“We have benchmarked the most widely used SAM-based bioimage annotation tools: SAMJ, Micro-SAM, and the SAM extension for QuPath. Each of these three tools serves a distinct niche, and their suitability depends on the user’s requirements. Tables A.2 and A.3 summarize aspects related to ease of use (available models, CPU-only compatibility, optional GPU acceleration, model-loading time, prompt-response speed, and one-click installation). Table A.4 reports the intersection-over-union (IoU) of annotations generated by each tool across multiple datasets.

Importantly, our benchmarking targets annotation performance, not full image segmentation. Annotation requires the tool to correctly identify and delineate an object given a prompt—that is, the user (or, in our evaluation, the ground truth) indicates where the object is, and the tool produces the corresponding mask. To evaluate annotation accuracy, we generated prompts directly from the ground-truth labels, produced annotations in each software using those prompts, and then compared the resulting masks with the ground-truth masks.

As a final remark, we note that QuPath-SAM and Micro-SAM also provide access to the original SAM models trained on natural images. To keep the comparison focused and the tables concise, we restricted our evaluation to the fine-tuned variants, which are expected to perform more reliably on cellular datasets. We additionally include SAM Huge in our benchmarking, as this model is available on Micro-SAM but not in SAMJ.”

Software	One-Click Installation	CPU Support	GPU Support	Models Available
SAMJ	YES	YES	NO	SAM2 Tiny
	YES	YES	NO	SAM2 Small
	YES	YES	NO	SAM2 Large
	YES	YES	NO	EfficientSAM
	YES	YES	NO	EfficientViTSAM-L2
Micro-SAM	NO	Slow	YES	SAM Base
	NO	Slow	YES	SAM Base Light
	NO	Slow	YES	SAM Base LM fine-tuned
	NO	Slow	YES	SAM Large LM fine-tuned
	NO	Slow	YES	SAM Large EM fine-tuned
	NO	YES	YES	MobileSAM
	NO	YES	YES	MobileSAM LM fine-tuned
	NO	YES	YES	MobileSAM EM fine-tuned
QuPath–SAM	NO	NO	YES	SAM Huge LM fine-tuned
	NO	NO	YES	SAM Huge EM fine-tuned
	NO	Slow	YES	SAM Huge
	NO	Slow	YES	SAM Large
	NO	Slow	YES	SAM Base
	NO	YES	YES	MobileSAM
	NO	YES	YES	SAM2 Tiny
	NO	YES	YES	SAM2 Small
	NO	YES	YES	SAM2 Base Plus
	NO	YES	YES	SAM2 Large

Table A.2 Comparison of features across SAM-based annotation tools.

Legend: LM = Light Microscopy, EM = Electron Microscopy

Software	Model	Load time (s)	Annotation time (s)
SAMJ	SAM2 Tiny	1.91	0.06
	SAM2 Small	2.00	0.07
	SAM2 Large	3.56	0.07
	EfficientSAM	2.69	0.08
	EfficientViTSAM-L2	1.37	0.08
Micro-SAM	MobileSAM LM	2.27	0.04
	MobileSAM EM	1.98	0.03
	SAM Base LM	8.65	0.05
	SAM Base EM	8.38	0.07
	SAM Large LM	26.03	0.06
	SAM Large EM	25.03	0.05
	SAM Huge	39.39	0.04
QuPath–SAM	SAM2 Tiny	NA	0.31
	SAM2 Small LM	NA	0.32
	SAM2 Base Plus EM	NA	0.58
	MobileSAM LM	NA	0.25
	SAM Huge EM	NA	Only GPU
	SAM Huge LM	NA	Only GPU

Table A.3 CPU-only model loading and annotation times across software tools. Load time denotes the time required to load a model and encode the image on the CPU before annotation begins. For QuPath–SAM, this metric is not applicable because the software does not pre-encode the image; instead, it encodes only the region around the user-provided prompt. If subsequent prompts fall within an already encoded region, no additional computation is required; otherwise, QuPath–SAM performs a new local encoding step.

Software	Model	[16] C	[22] C	[23] B	[24] C	[25] N	[25] C	[26] M	[27] M
SAMJ	SAM2 Tiny*	0.64	0.35	0.36	0.57	0.66	0.45	0.79	0.69
	SAM2 Tiny†	0.77	0.74	0.74	0.84	0.81	0.72	0.88	0.82
	SAM2 Small*	0.63	0.35	0.36	0.57	0.67	0.42	0.82	0.71
	SAM2 Small†	0.77	0.73	0.74	0.84	0.82	0.73	0.88	0.82
	SAM2 Large*	0.66	0.37	0.42	0.60	0.70	0.43	0.82	0.73
	SAM2 Large†	0.78	0.74	0.73	0.84	0.81	0.72	0.88	0.82
	EfficientSAM*	0.63	0.40	0.39	0.55	0.68	0.42	0.69	0.70
	EfficientSAM†	0.74	0.69	0.68	0.81	0.77	0.62	0.87	0.79
	EfficientViTSAM-L2*	0.69	0.50	0.41	0.61	0.71	0.47	0.82	0.73
	EfficientViTSAM-L2†	0.80	0.77	0.76	0.86	0.85	0.76	0.90	0.86
Micro-SAM	MobileSAM LM*	0.69	0.60	0.60	0.77	0.75	0.66	0.33	0.65
	MobileSAM LM†	0.82	0.80	0.76	0.85	0.84	0.80	0.77	0.84
	MobileSAM EM*	0.48	0.47	0.40	0.60	0.67	0.56	0.39	0.76
	MobileSAM EM†	0.62	0.71	0.62	0.78	0.87	0.77	0.79	0.87
	SAM Base LM*	0.74	0.69	0.70	0.82	0.74	0.70	0.46	0.69
	SAM Base LM†	0.85	0.83	0.82	0.89	0.84	0.82	0.79	0.84
	SAM Base EM*	0.58	0.49	0.38	0.58	0.64	0.53	0.53	0.81
	SAM Base EM†	0.77	0.71	0.65	0.76	0.84	0.78	0.80	0.88
	SAM Large LM*	0.77	0.71	0.74	0.83	0.75	0.71	0.49	0.69
	SAM Large LM†	0.86	0.83	0.83	0.89	0.84	0.83	0.78	0.84
	SAM Large EM*	0.57	0.50	0.41	0.57	0.62	0.53	0.59	0.82
	SAM Large EM†	0.76	0.69	0.63	0.75	0.84	0.78	0.78	0.89
	SAM Huge*	0.64	0.28	0.30	0.55	0.64	0.35	0.44	0.70
	SAM Huge†	0.76	0.69	0.62	0.75	0.79	0.59	0.71	0.82
QuPath–SAM	SAM2 Tiny*	0.57	0.29	0.32	0.57	0.28	0.36	0.41	0.75
	SAM2 Tiny†	0.74	0.71	0.65	0.78	0.72	0.62	0.65	0.84
	SAM2 Small*	0.56	0.32	0.32	0.55	0.25	0.34	0.42	0.70
	SAM2 Small†	0.76	0.68	0.64	0.77	0.75	0.65	0.65	0.84
	SAM2 Base+*	0.59	0.40	0.31	0.55	0.25	0.38	0.42	0.59
	SAM2 Base+†	0.75	0.70	0.63	0.76	0.71	0.64	0.65	0.84
	MobileSAM*	0.47	0.25	0.31	0.54	0.20	0.27	0.25	0.51
	MobileSAM†	0.71	0.62	0.61	0.73	0.68	0.57	0.64	0.77
	SAM Huge LM*	0.66	0.69	0.70	0.77	0.45	0.53	0.29	0.67
	SAM Huge LM†	0.75	0.80	0.81	0.87	0.68	0.64	0.69	0.73
	SAM Huge EM*	0.59	0.45	0.46	0.56	0.48	0.51	0.46	0.81
	SAM Huge EM†	0.74	0.65	0.70	0.72	0.63	0.68	0.71	0.80

Table A.4 IoU of annotations produced by each model across datasets. For each software tool and model variant, we report the IoU between the predicted masks and the ground-truth annotations on the following benchmark datasets: Cellpose [16], LiveCell [22], DeepBacs [23], NeurIPS Cell Segmentation [24], TissueNet Nuclei [25], TissueNet Cells [25], MitoEM [26] and CEM-MitoLab [27]. Importantly, this evaluation measures *annotation performance*, not full-image segmentation: prompts were automatically derived from the ground-truth labels and each tool generated a corresponding mask, which we compared directly to the ground truth. For conciseness, in Micro-SAM and QuPath-SAM we evaluated only the fine-tuned SAM variants and SAM Huge. The fine-tuned models are expected to perform best on cellular data, and SAM Huge is not available in SAMJ.

Legend: * 3-point multipoint prompt; † bounding box prompt; (C) cells; (B) bacteria; (N) nuclei; (M) mitochondria; (LM) fine-tuned on light microscopy images; (EM) fine-tuned on electron microscopy images.

Comment 1.2. User Feedback: Consider including results or a short discussion from a preliminary user survey or usability evaluation, particularly with non-technical biologists.

Author answer:

We agree that user experience is a key aspect of SAMJ's value and have expanded the manuscript to clarify how usability support is provided. While we did not run a formal survey during manuscript preparation, we have added a concise **"Usability and troubleshooting"** section in the Online Methods. This section directs users to the documentation, outlines basic troubleshooting steps, advises users to submit questions via GitHub or on the Image.sc Forum if they encounter obstacles, and links to explanatory tutorial videos.

As additional context, SAM models and SAMJ have been demonstrated multiple times in workshops and community events, where informal feedback has been consistently positive: for example, during the I2K 2024 conference and the EuroBioImaging Image Data Community Days 2025. In the Imaging Lunch workshop at EPFL, more than 50 PhD students from various fields were able to install and run SAMJ without any difficulties across a wide range of computer configurations. Likewise, at the EMBO Practical Course on Light Sheet Microscopy, approximately 15 researchers used SAMJ successfully during hands-on sessions. These experiences suggest that the tool is accessible even to non-technical users and works reliably across platforms.

Author changes, added section in Online Methods, line 395:

"*SAMJ was designed with accessibility and usability in mind, but we recognize that new users may still face challenges. To facilitate onboarding, we provide a set of short demo videos in the Supplementary Materials, which offer a quick start for most use cases.*

Because SAM is a computationally demanding model, we incorporated several SAM variants so that users can select an option suitable for their hardware. On computers with limited resources, loading a large model may take several minutes, which can be mistaken for a crash. In such cases, we recommend starting with lighter variants (e.g., EfficientViT-L2 or EfficientSAM) and then moving to larger models if performance allows. For reference, all development was carried out on a Linux workstation with an Intel i7 CPU and 32 GB RAM, where models typically load in under 6 seconds. Even on an older 10-year-old Intel i3 with 16 GB RAM, SAM2 Small loaded in under two minutes.

Users should also note that the first model load and the first annotation after installation or system startup are slower than subsequent runs, since Python needs to cache required libraries. For example, the first load of SAM2 Tiny may take 1–2 minutes and the first annotation up to 7–9 seconds, while subsequent operations are nearly instantaneous.

Each model is installed in its own Python environment, stored in the Appose directory under the Fiji/ImageJ folder. Although these environments can be accessed directly in Python, we recommend not modifying them, as changes may break SAMJ's configuration. If an environment becomes inconsistent (e.g., missing libraries or version mismatches), SAMJ will

prompt reinstallation, which replaces the old environment, deleting the user's work if they had done any there.

Finally, for troubleshooting or suggestions, users are encouraged to post questions on the Image.sc forum or to open an issue on GitHub (https://github.com/segment-anything-models-java/SAMJ-IJ/issues)."

Comment 1.3. Documentation & Stability: Please comment briefly on the long-term maintenance plans, especially for keeping compatibility with evolving versions of SAM and Fiji.

Author answer:

The reviewer raises an important point regarding long-term maintenance and compatibility, which we have carefully considered in the system design. SAMJ is engineered for stability and robustness. The current release keeps the Java side deliberately simple with few dependencies and benefits from Java's mature, stable runtime. Through its integration with Appose, SAMJ executes all Python components in an isolated environment with **pinned package versions.** This architecture decouples SAMJ from upstream Fiji updates and shields it from changes in the Python ecosystem, ensuring consistent behaviour over time.

For the long term, we will maintain compatibility with new SAM-family variants, track the update cycle of Fiji via continuous tests, and provide versioned releases and documentation on GitHub. Community feedback through GitHub and the Image.sc forum will guide priorities, ensuring that SAMJ remains aligned with both the ecosystem of Fiji and the evolving SAM landscape.

Comment 1.4. Batch Mode Flexibility: While BatchSAMize is a valuable feature, clarifying its extensibility to more complex prompt types or segmentation goals could enhance reproducibility.

Author answer:

We acknowledge the reviewer's suggestion to clarify the extensibility of the *BatchSAMize* feature and have expanded the corresponding section accordingly. We have clarified at line 133 in the manuscript that **BatchSAMize** accepts prompts originating from manual drawing, Fiji commands, other plugins, or DL models, or import via ROI Manager. This design strongly supports reproducibility, since lists of prompts and all preprocessing steps can be saved, shared, and read again and re-run identically via macros/scripts.

Authors changes, line 133:

"*Prompts for BatchSAMize can be supplied either manually or automatically. Thanks to SAMJ's integration with Fiji, users can combine bounding boxes or point prompts generated by deep learning models, macros, or files imported through the ROI Manager. Users can even use the macros to annotate batches of images with SAMJ. This flexibility enhances both the reproducibility and the automation of image annotation.***"**

Author changes, added section in Supplementary Materials, line 432:

"SAMJ supports macro usage via BatchSAMize with preset prompts. Here we show three examples demonstrating how to integrate SAMJ into ImageJ workflows using macros.

Single-prompt example

The following macro creates a single-point prompt in ImageJ and annotates it with SAMJ.

```
// Open example image
run("Blobs (25K)");

// Use the "multipoint" tool and create a prompt
makePoint(177, 33, "small yellow hybrid");

// Use SAM2 Tiny to annotate the selected object.
// This command also generates a label mask.
run("SAMJ Annotator", "model=[SAM2 Tiny] export=true");
```

Automatic prompts using Find Maxima

This macro uses ImageJ's Find Maxima command to automatically generate prompts for all detected objects. SAMJ then annotates each instance.

```
// Open example image
run("Blobs (25K)");

// Invert the image so objects are white and the background is black
run("Invert");

// Use ImageJ Find Maxima to detect one prompt per instance
run("Find Maxima...", "prominence=100 output=[Point Selection]");

// Use SAM2 Tiny to annotate all detected prompts.
// This command also generates a label mask.
run("SAMJ Annotator", "model=[SAM2 Tiny] export=true");
```

Processing all images in a folder

This macro processes every image inside a folder.
For each image, it detects instance-level prompts via Find Maxima and annotates them with SAMJ.

```
// Set the directory that contains all the images you want to annotate
directory = "/path/to/images/folder";
files = getFileList(directory);

for (i = 0; i < files.length; i++) {
      // Open each of the images of the folder
```

```
        open(directory + File.separator + files[i]);
        selectImage(files[i]);

        // Use ImageJ Find Maxima to detect one prompt per instance
        run("Find Maxima...", "prominence=100 output=[Point Selection]");

    // Use SAM2 Tiny to annotate all detected prompts.
    // This command also generates a label mask.
    run("SAMJ Annotator", "model=[SAM2 Tiny] export=true");
}“
```

Reviewer 2's comments:

The article is generally well-written and provides a concise overview of the potential applications for SAMJ. Harnessing SAM for streamlining image annotation and segmentation, especially for complex structures found in cells and tissue has important implications for the field of biomedical science. The authors make several claims about the advantages of SAMJ, mostly relating to the acceleration and precision (page 7, lines 190-191) of user-driven annotation. Additionally, several other AI-assisted tools and plugins were mentioned throughout the manuscript.

We thank Reviewer 2 for their careful reading and constructive remarks. Their comments helped us substantially strengthen the manuscript, especially to clarify SAMJ's position within the broader SAM-based ecosystem. The points raised prompted us to expand the evaluation presented in the paper by adding a comprehensive benchmarking section, which we believe now strengthens the position of SAMJ. We appreciate the reviewer's attention to usability, model comparison, and domain applicability, and we address each of these aspects in detail in the responses below.

Comment 2.1. This study would greatly benefit by substantiating these claims through conducting a more systematic evaluation (e.g., speed, accuracy, robustness metrics) of SAMJ compared to other available tools. It would be helpful for the reader to understand how SAMJ objectively performs, how it offers tangible benefits to the end-user, and its limitations.

Author answer:

We appreciate the reviewer's suggestion regarding the value of a broader evaluation of SAM. Initially, we chose not to include benchmarking in order to keep the manuscript focused on the software contribution rather than on model performance. However, after careful consideration, and thanks to Reviewer 2's constructive recommendation, we conducted a detailed comparative evaluation of SAMJ alongside the main SAM-based tools currently available for bioimage analysis, to our knowledge: Micro-SAM (napari) and the SAM extension for QuPath. Our goal is not to position any tool as superior, but rather to provide transparent evaluation showing that these tools perform comparably and excel in

different usage contexts. This evaluation allows readers to choose the tool best suited to their workflow and hardware constraints.

We have added a new section to the manuscript with the detailed results and benchmarking done for SAMJ and the two other softwares. In this section, we provide three tables: the first compares general features of the software (one-click installation, GPU/CPU, available models), the second table measures the loading and prompting time of each of the software and the last table benchmarks the annotation Intersection-over-Union of each of the models of each of the software on a series of microscopy datasets. It is important to note that this evaluation measures annotation performance, not full-image segmentation: prompts were automatically generated from the ground-truth labels, and each tool produced a mask in response to these prompts, which we then compared to the ground truth.

Author changes, added section in Online Methods, line 261:

See response to Reviewer 1 to Comment 1.1

Comment 2.2. The authors mention that "SAM has been made available in commonly used bioimage software packages" (page 2, line 58). Has SAM been integrated with other tools already and if so, how is SAMJ differentiated from them? For example, there appears to be other SAM adaptations that have been described in the field, such as 'MedSAM' (Ma et al., 2024).

Author answer:

We thank the reviewer for this opportunity to clarify SAMJ's position within the current software landscape. While we acknowledge that SAM integrations exist for platforms like napari and QuPath, SAMJ differentiates itself through its deep integration with ImageJ/Fiji, the most widely adopted bioimage analysis ecosystem. With SAMJ users can leverage SAM models with Fiji's rich plugin ecosystem, ROI tools, and macro/scripting capabilities.

Beyond standard integration, SAMJ offers unique functionality designed specifically for the Fiji user base. Most notably, its macro-aware design enables reproducible automatic prompting and the execution of the 'BatchSAMize' mode to process large image sets, capabilities that are highly requested by users but generally unavailable in other GUI-based SAM tools. Furthermore, SAMJ significantly lowers the barrier to entry by managing the Python environment internally; to our knowledge, it is the only tool that provides access to SAM without requiring any command-line interaction, facilitating its adoption by non-computational researchers.

To broaden access, SAMJ ships with **five model variants** spanning different computational footprints and performance profiles, allowing users to match the model to their hardware, from high-end workstations to modest laptops.

Finally, as said in the previous point, we have added a new section in the Online Methods where we highlight the strengths of each of the SAM wrappers and showcase the unique features that SAMJ brings to the table.

Regarding domain-specific, fine-tuned SAM derivatives (e.g., MedSAM for medical imaging and micro-SAM for microscopy), we have not bundled these at this time. SAMJ currently focuses on general-purpose variants trained on natural images; however, its modular design leaves the door open to include additional models in future releases as community demand or superior options emerge.

Comment 2.3. Page 4, lines 97-100: the article states that “five SAM variants are available through SAMJ”, yet only three are mentioned in the paragraph. What are these variants and what do they do?

Author answer:

We have corrected and made explicit the five supported variants: SAM-2 Tiny, Small, and Large; EfficientSAM; and EfficientViTSAM-l2, and summarized the trade-offs between resource use and annotation performance. We intentionally include this spectrum of models to maximize accessibility. Each variant has distinct computational requirements and performance characteristics, enabling users to select an option that fits their available hardware and accuracy/speed needs. We also point readers to model sizes and encoding times (Appendix A.3, Table A.5) and provide guidance on choosing a variant based on object complexity and hardware.

Author changes, line 109:

“In addition, SAMJ provides access to five SAM variants: SAM-2 (Tiny, Small, and Large), EfficientSAM, and EfficientViTSAM-L2. All variants perform the same segmentation task and are trained on comparable generalist datasets, but they differ substantially in computational requirements and performance characteristics (see Table A.5). This diversity enables users to select a model that fits both their hardware and application needs, ensuring that at least one option remains practical to run on any virtual-machine, workstation or mid-range laptop. For instance, users may prioritize speed and efficiency on less powerful machines or higher annotation accuracy on complex images.”

Comment 2.4. It is described that SAMJ offers two annotation modes – Live and Batch (page 2, line 108). In Batch mode, can you define seed points across different images? Can BatchSAMize be used to batch annotate a set of images?

Author answer:

We thank the reviewer for this insightful question regarding the use of *BatchSAMize* across multiple images. Because SAMJ is fully integrated within the Fiji ecosystem, users can leverage Fiji’s macro language to iterate through folders of images and apply *BatchSAMize* sequentially. This enables batch annotation across multiple datasets while maintaining reproducibility and flexibility. We have added a brief note about this in the main text and included a section about macros in the *Supplementary Materials* to illustrate this approach.

Author changes, line 133:

“Prompts for BatchSAMize can be supplied either manually or automatically. Thanks to SAMJ’s integration with Fiji, users can combine bounding boxes or point prompts generated by deep learning models, macros, or files imported through the ROI Manager. Users can even use the macros to annotate batches of images with SAMJ, as illustrated in

subsection B.2. This flexibility enhances both the reproducibility and the automation of image annotation."

Author changes, added section in Supplementary Materials, line 432:

See response to Reviewer 1 to Comment 1.4

Comment 2.5. Figure 2 highlights specific use cases to demonstrate the versatility of SAMJ applications. See below for questions regarding specific applications:

a. Can the authors demonstrate the use of SAMJ for segmenting subcellular structures (i.e. organelles) or cells with more complex morphologies (i.e. neurons)? The authors mention in page 6, line 135 that "elongated or branched structures may pose more of a challenge". Can you speculate why this is? Is this an inherent limitation of the SAM foundational DL model?

Author answer:

We agree that this is an important question that concerns more the limitations of the SAM foundational model than of SAMJ itself. As SAMJ serves as an interface to run and visualize SAM-family models within Fiji, it reproduces the segmentation output of SAM without post-processing or model modification. The manuscript already includes a 3D EM use case (mitochondria) illustrating subcellular annotation. In general, elongated or highly branched structures such as neurons remain challenging for promptable segmenters trained primarily on natural-image statistics, due to scale and continuity ambiguities. These limitations have been discussed in the original SAM and SAM2 publications, to which we refer the reviewer for further details.

Nevertheless, SAMJ mitigates these model-inherent limitations by enabling a robust **human-in-the-loop workflow**. Through real-time feedback, users can interactively stitch together complex biological structures; for example, users can segment highly branched or elongated objects by placing multiple prompts along different extensions and iteratively refining the mask to ensure continuity. We have clarified these aspects in a new *Online Methods* section.

Author changes, added section in Online Methods, line 382:

"*The Segment Anything Model was originally trained with a one-prompt/one-object paradigm on a wide variety of objects, mostly from natural images. As a result, biological structures with intricate morphologies, such as neurons, are often difficult to annotate with a single prompt, regardless of prompt placement.*

However, thanks to SAMJ interactivity, users can overcome this limitation by enabling a multi-step, compositional annotation workflow. While SAM may struggle to segment a branched structure globally, it remains highly effective at detecting local boundaries. SAMJ leverages this strength by allowing users to segment distinct sub-structures (e.g., soma, axons, and dendrites) individually. These partial masks can then be merged into a single, coherent object. This piecewise strategy yields significantly more accurate annotations for elongated or highly branched structures than single-prompt inference (see Figure A.3)."

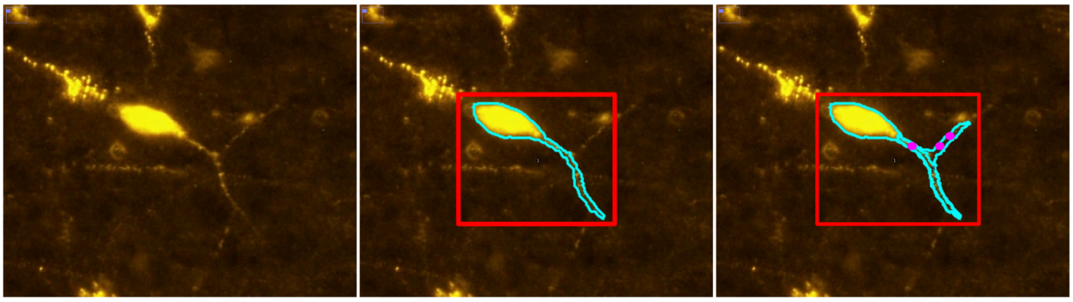

Figure A.3 **Multi-step multi-prompt single object annotation** SAMJ's interactive workflow allows users to segment multiple parts of an object that the model recognizes and subsequently merge them. The figure shows a fluorescent neuron [28] with two ramifications. Using a single box prompt (middle image), the model captures only one ramification. By annotating the second ramification with additional point prompts (purple dots on the right image), the user obtains two separate labels whose union yields the final mask of the complex neuron.

b. In addition to tumor tissue, can SAMJ be used to automatically annotate complex tissue, such as brain regions?

Author answer:

We thank the reviewer for the question and are happy to see them pondering scenarios where SAMJ could be useful. While our manuscript primarily focuses on providing the bioimage analysis community with a tool that integrates the capabilities of the SAM model for efficient image annotation, we emphasize that SAMJ inherits both the strengths and limitations of the underlying SAM model and its variants.

The use cases included in the manuscript are solely intended to demonstrate how SAMJ functions in practice, rather than to comprehensively evaluate the model's performance across diverse tissue types. As such, the generalization ability of SAMJ to other complex tissues depends largely on the SAM model itself, and further domain-specific validation would be required. Our contribution lies in offering an accessible and extensible annotation framework within Java-based platforms.

We clarify that SAM is agnostic to tissue type and can be applied to complex tissues; masks can then be combined with downstream Fiji pipelines (e.g., region quantification).

c. The authors state that 3D segmentation is challenging in general. Can more details be shared on why SAMJ was successful in the mitochondrial use-case? How many slides does the user need to annotate for SAMJ to reliably segment mitochondrial within the entire tissue volume in 3D?

Author answer:

We augmented the Use Case explanation to state explicitly that in the showcased 3D EM example, we annotated **every slice** (a pragmatic 2.5D strategy), leveraging SAMJ's rapid interactive prompts to ensure spatial consistency across slices. We have added a clarification for this in the manuscript.

Author changes, line 191:

"In this example, every slice of the volume was annotated with SAMJ, effectively following a pragmatic 2.5D strategy. Thanks to SAMJ's rapid interactive prompting, this approach still provided a substantial gain in speed compared to fully manual slice-by-slice annotation."

Comment 2.6. From a user-accessibility standpoint, can the authors provide further commentary on troubleshooting tips or suggestions to address most common hurdles when using SAMJ?

Author answer:

We added a brief "**Usability and troubleshooting**" paragraph in the Online Methods that centralizes links to documentation, demo videos, and support channels (GitHub issues and Image.sc).

Author changes, added section in Online Methods, line 395:

See response to Reviewer 1 to Comment 1.2

Comment 2.7. Relevant information regarding model comparison, performance and evaluation are found within the Appendix section. It would have been helpful to explicitly reference these supplementary tables and figures from the main text. Addressing this issue may in part, answer some of my comments above.

Author answer:

We agree and have added explicit cross-references to Appendix figures/tables wherever they are relevant in the main text.

Response to Reviewers

We sincerely thank the reviewers for their careful and constructive evaluation of our revised manuscript. We greatly appreciate their positive assessment and helpful suggestions, which have further improved the clarity and quality of the paper. Below, we address the final comments.

Answer to Reviewer 1 comments

1. Minor suggestion:

In a few places, qualitative statements such as “visually indistinguishable from ground truth” (e.g., bacterial motility use case) could be slightly strengthened by explicitly referencing the corresponding quantitative results already reported (e.g., IoU values in Table A.4), or by explicitly stating that these observations are qualitative.

Answer:
Good point. We have changed the wording, pointing at the IoU benchmark table

Author changes , line 190:
“*When compared to manually generated ground truth, SAMJ’s annotations are highly consistent with the reference labels, as supported by the IoU results reported in Table 4.”*

2. Minor suggestion:

A brief explicit mention of limitations in the main text (not only in the appendices) for example, challenges with highly elongated or extremely thin structures, or dependence on prompt quality would further improve transparency.

Answer:
We have now moved the mentions to the dependence on prompt quality dependence and challenges to the Methods section of the article

Author changes , section Review of SAM-Based Annotation Tools for Bioimaging moved to main text, line 278:

“*We have benchmarked the most widely used SAM-based bioimage annotation tools: SAMJ, Micro-SAM, and the SAM extension for QuPath. Each of these three tools serves a distinct niche, and their suitability depends on the user’s requirements. Table 2 and Table 3 summarize aspects related to ease of use (available models, CPU-only compatibility, optional GPU acceleration, model-loading time, prompt-response speed, and one-click installation). Table 4 reports the intersection-over-union (IoU) of annotations generated by each tool across multiple datasets.*

Importantly, our benchmarking targets annotation performance, not full image segmentation. Annotation requires the tool to correctly identify and delineate an object given a prompt—that is, the user (or, in our evaluation, the ground truth) indicates where the object is, and the tool produces the corresponding mask. To evaluate annotation accuracy, we generated prompts directly from the ground-truth labels, produced annotations in each software using those prompts, and then compared the resulting masks with the ground-truth masks.

As a final remark, we note that QuPath-SAM and Micro-SAM also provide access to the original SAM models trained on natural images. To keep the comparison focused and the tables concise, we restricted our evaluation to the fine-tuned variants, which are expected to perform more reliably on cellular datasets. We additionally include SAM Huge in our benchmarking, as this model is available on Micro-SAM but not in SAMJ."
